# Characterizing the Diffusion Properties of Prostate Tissue Using Paired MR Microscopy and Multidimensional Diffusion MRI

**DOI:** 10.1002/mrm.70344

**Published:** 2026-03-24

**Authors:** Adam Phipps, Nyoman D. Kurniawan, Geoff Watson, Paul Sved, Samson Dowland, Eleftheria Panagiotaki, David Atkinson, Roger Bourne

**Affiliations:** ^1^ Centre for Medical Imaging, Division of Medicine University College London London UK; ^2^ Australian Institute for Bioengineering and Nanotechnology, Centre for Advanced Imaging The University of Queensland Brisbane Queensland Australia; ^3^ New South Wales Health Pathology Sydney New South Wales Australia; ^4^ School of Medical Sciences, Faculty of Medicine and Health University of Sydney Sydney New South Wales Australia; ^5^ Hawkes Institute University College London London UK; ^6^ Sydney School of Health Sciences, Faculty of Medicine and Health University of Sydney Sydney New South Wales Australia

## Abstract

**Purpose:**

To characterize the diffusion properties of prostate epithelium and stroma in benign tissue and cancer.

**Methods:**

Paired MR microscopy (20 μm) and multidimensional diffusion MRI (dMRI) (160 μm, *b* = 1000–2000 s/mm^2^, ∆ = 15–120 ms) were performed at 16.4 T on 17 fixed prostate tissue samples (14× benign, 2× Gleason 3 + 3, 1× Gleason 4 + 4). MR microscopy images were used to segment epithelial, stromal, and luminal components in each sample. For each dMRI sequence, aggregate epithelial and stromal signal contributions in benign tissue were estimated using a linear epithelium‐stroma‐lumen signal model. Four diffusion signal models were fit to these aggregate signals. Quality‐of‐fit was assessed using the small‐sample corrected Akaike Information Criterion (AICc). Voxel‐wise model fitting was also performed to compare parameter estimates in benign tissue and cancer.

**Results:**

Aggregate dMRI signal estimates for both epithelium and stroma were best described by the Ball + Sphere model (lowest AICc). A higher sphere fraction (0.278 vs. 0.175) and lower ball‐compartment diffusivity (0.611 vs. 0.943 μm^2^/ms) were estimated for epithelium compared to stroma. The ADC model provided the worst fit in both cases (highest AICc). For Gleason 3 + 3 cancer, ADC and Ball + Sphere parameter estimates were consistent with the values found for benign epithelium and stroma; however, raised sphere fraction estimates were seen in Gleason 4 + 4 cancer.

**Conclusion:**

Direction‐averaged diffusion in fixed prostate epithelium and stroma is well described by the Ball + Sphere model. The diffusion properties of epithelium in Gleason 3 + 3 cancer and benign tissue appear to be similar; however, a marked increase to the volume fraction of restricted water was found for Gleason 4 + 4 epithelium.

## 1 Introduction

Currently, prostate cancer diagnoses rely on histological assessment of biopsy samples, where cancer is graded based on the observed glandular microstructure [1]. However, the biopsy procedure is invasive, carries risk of complications [2], and may return an inaccurate assessment of clinical significance given only very small volumes of tissue are assessed (< 200 mm^3^ and < 1% of prostate volume [3]). Noninvasive methods for prostate cancer diagnosis and grading are desirable.

Diffusion MRI (dMRI) is non‐invasive, sensitive to tissue microstructure, and enables assessment of whole prostate volumes, so is a promising source of prostate cancer biomarkers [4, 5, 6]. At present, dMRI plays an important role in the detection and management of prostate cancer; however, clinical use (multiparametric MRI) is limited to qualitative assessment of high‐*b*‐value images and ADC maps [7]. Clinical significance is often overestimated through multiparametric MRI, leading to high biopsy referral rates with many biopsies turning out negative [8].

There is an ongoing research focus on quantitative dMRI techniques that use mathematical signal models to measure the diffusion properties of prostate tissue [9]. The measurements obtained through these techniques could act as quantitative biomarkers for prostate cancer, which may enable more reliable tracking of disease progression, reduce the subjectivity associated with qualitative image assessment, and potentially improve the accuracy of imaging‐based grading.

An important stage in establishing measurements as meaningful biomarkers for disease is “biological validation,” which refers to the stepwise linking of measurements to disease biology [10]. Understanding how the biological changes of prostate tissue across Gleason grades influence dMRI measurements will help assess the potential for non‐invasive prostate cancer grading using dMRI. However, the biological foundation of current techniques is limited; the relationships between measurements and disease biology are mainly speculative and require more thorough validation.

Prostate tissue is composed of three main components: epithelium, stroma, and lumen (ESL) [11]. Epithelium refers to the cells that line prostatic gland acini, stroma is a fibromuscular connective tissue that surrounds the glands, and lumen refers to the fluid‐filled space within each gland acinus. Prostate adenocarcinoma is the most common form of prostate cancer and is associated with the proliferation of epithelial cells, with invasive growth of epithelium into luminal space and stroma [12]. Higher‐numbered Gleason patterns are characterized by the loss of glandular differentiation and reduction in stromal volume, and loosely correlate with the local volume fractions of epithelium, stroma, and lumen.

The Hybrid Multidimensional MRI (HM‐MRI) technique [6] tries to quantify the ESL volume fractions within each voxel, and uses the volume fractions of epithelium and lumen to form a biomarker for cancer. This technique assumes that the diffusion and relaxation properties of epithelium do not change with Gleason pattern; however, the validity of this assumption has not been verified. Other methods, such as ADC, Kurtosis [13], “Vascular, Extracellular, and Restricted Diffusion for Cytometry in Tumours” (VERDICT [4]), and time‐dependent diffusion MRI [14] use signal models that are not specific to the ESL tissue components, and aim to distinguish benign tissue and cancer through changes to estimated model parameters. In these cases, it is important to understand the influence of the biological changes between Gleason patterns on model parameters, as this will help to evaluate the feasibility of cancer grading using modeling results.

Previous studies have used direct MRI‐histology comparisons to characterize the relationships between dMRI measurements and tissue microstructure [15, 16, 17, 18, 19]; however, achieving accurate registration and slice alignment between MRI and histology images is very challenging, histology sections (˜5 μm thick) only represent a tiny fraction of the tissue within an MRI voxel, and histology is destructive, so repeat experiments are not possible [20]. As an alternative, we aim to use MR microscopy images to form “ground truth” maps of tissue microstructure and evaluate dMRI measurements. This approach avoids registration challenges, since MR microscopy and dMRI images can be acquired in exact spatial alignment, and enables repeat experiments, as microscopy imaging is nondestructive.

In this work, we combine MR microscopy, multidimensional dMRI, and diffusion signal modeling to characterize the diffusion properties of benign and malignant prostate tissue. The results from this work will help to assess the potential for non‐invasive prostate cancer grading using quantitative dMRI.

## 2 Methods

### 2.1 Prostate Tissue Samples

Seventeen cylindrical cores of prostate tissue (approx. 3 mm diameter × 4 mm length) were extracted from macro slices of three formalin‐fixed radical prostatectomy specimens. Tissue cores were extracted using a 3 mm diameter biopsy punch at sites selected by a specialist uropathologist to provide a diversity of tissue types at locations that would not affect clinical reporting results. Samples were collected with institutional research ethics approval and informed patient consent.

Samples were stored in 10% neutral‐buffered formalin for 3–7 days. Given the small size of samples, this duration was sufficient to ensure complete formalin penetration and tissue fixation [21].

Prior to imaging, tissue cores were equilibrated overnight in a phosphate‐buffered solution containing 0.5 mL/L Magnevist (Bayer) gadolinium, then were inserted into 4 mm o.d. 30 mm long Polytetrafluoroethylene (PTFE) tubes with 2–3 samples per tube. To secure the samples, liquid gelatin was injected into the PTFE tubes and allowed to set. Each PTFE tube was then mounted in a separate 5 mm o.d. NMR tube filled with the Magnevist solution.

### 2.2 Imaging

#### 2.2.1 Magnetic Resonance Imaging

Magnetic resonance imaging was performed using a 700 MHz (16.4 Tesla) Bruker system equipped with a Micro5 probe and running ParaVison version 6.0.1. One NMR tube (2–3 samples) was imaged per session. The ambient temperature during imaging was 22°C–23°C.

An initial multi‐slice multi‐echo (MSME) sequence was performed for inspection of the samples and T2 measurement. The results from T2 calculation are included in the Supporting Information (Section S2).

Following this, imaging included two components: MR microscopy, including a multi‐gradient echo (MGE) sequence [22] (20 μm isotropic resolution, TE = 16–34 ms) and DTI sequence (40 μm isotropic, *b* = 1200 s/mm^2^, 6 directions); and multidimensional dMRI (160 μm isotropic resolution), including 10 stimulated‐echo acquisition mode [23] (STEAM) sequences, with five *b*‐values (1000–2000 s/mm^2^) and two diffusion times per *b*‐value (Δ^−^ = 15–50 ms, Δ^+^ = 40–120 ms). Multidimensional dMRI was acquired at lower resolution to maintain a practical total scan time, and a STEAM sequence was used to enable imaging at longer diffusion times without excessive T2 decay. Additionally, a *b* = 0 spin echo sequence was acquired at 160 μm isotropic resolution for dMRI signal normalization. All sequence details are provided in Table 1.

**TABLE 1:** MRI sequence parameters for all imaging sequences.

Sequence name	TE (ms)	TR (ms)	δ/∆ (ms)	*b* (s/mm^2^)	No. directions	Acquisition matrix size	Acquired resolution (μm^3^)	Field of view (FOV) (mm^3^)	Scan time
2D Multi‐slice multi‐echo (MSME)	8, 16, 24, …, 80 (10 echos)	1000	—	—	—	(2×) 1 × 256 × 256 (2 noncontiguous slices)	(2×) 200 × 19.5 × 58.6	(2×) 0.2 × 5.0 × 15.0	4 min
3D Multi‐Gradient Echo (MGE)	16, 22, 28, 34	80	—	—	—	240 × 240 × 640	20 × 20 × 20	4.8 × 4.8 × 12.8	1 h 49 min
3D Spin Echo Diffusion Tensor Imaging (DTI)	19	400	2/12	0, 1200	6	120 × 120 × 320	40 × 40 × 40	4.8 × 4.8 × 12.8	10 h 45 min
3D Spin Echo *b* = 0 (SE b = 0)	14	750	—	0	—	30 × 30 × 80	160 × 160 × 160	4.8 × 4.8 × 12.8	39 min
3D Stimulated Echo dMRI (STEAM dMRI)	14	750	2/15, 2/40; 2/20, 2/60; 2/30, 2/80; 2/40, 2/100; 2/50, 2/120	1000; 1250; 1500; 1750; 2000	6	30 × 30 × 80	160 × 160 × 160	4.8 × 4.8 × 12.8	1 h 9 min

*Note*: All sequences included conventional Cartesian readouts, acquiring one k‐space line per TR. The MGE, DTI, SE *b* = 0, and STEAM dMRI sequences were acquired in 3D. The MSME sequence was acquired in 2D. The DTI and dMRI sequences used the same six gradient directions. Directions were determined by the scanner using an electrostatic repulsion method (Jones DK et al. MRM, 2004). The same sequences were used for all sample sets.

#### 2.2.2 Histology

Following MRI, samples were prepared for optical microscopy using routine paraffin embedding, sectioning, and hematoxylin & eosin (H&E) staining methods. Axial sections were acquired at ≈250 μm intervals, resulting in 10–15 sections per sample.

Tissue sections were examined and reported by a specialist uropathologist (GW). Three samples containing cancer were identified: two samples from the first specimen containing Gleason grade 3 + 3 cancer, and one sample from the second specimen containing Gleason grade 4 + 4 cancer.

More detail on the samples is included in the Supporting Information (Section S1).

### 2.3 Analysis

The following provides a high‐level overview of the analysis presented in this work. All analyses were performed on magnitude imaging data.

The primary aims were to: (1) characterize the diffusion properties of epithelium and stroma in benign tissue and assess heterogeneity across the 14 benign tissue samples; and (2) characterize the changes in epithelial diffusion properties across the three samples containing cancer and compare changes to the baseline heterogeneity observed in benign tissue.

MR microscopy images were used to segment the epithelial, stromal, and luminal components of each sample (Section 2.3.1). Segmentation performance was evaluated against histology.

Multidimensional dMRI images were not acquired at high enough resolution to resolve ESL components, so the segmentations computed from MR microscopy were used to calculate the volume fractions of epithelium, stroma, and lumen within each dMRI voxel.

Each dMRI signal measurement was represented as a weighted linear sum of epithelial, stromal, and luminal signal contributions, with weights reflecting the volume fraction of each component within the voxel. For each dMRI sequence, this linear signal model was fit to the dMRI signal measurements across all voxels in benign tissue. This returned estimates for aggregate epithelial and stromal signal contributions across the benign tissue samples. The residuals from linear model fitting were analyzed to assess the heterogeneity of epithelial and stromal diffusion properties across the benign samples (Section 2.3.3).

Four dMRI signal models were fit to the aggregate signals estimated for benign epithelium and stroma to characterize and compare the diffusion properties of the two components (Section 2.3.4).

Finally, voxel‐wise model fitting was performed for all samples. Parameter estimates in benign tissue and cancer were compared (Sections 2.3.4 and 2.3.5).

#### 2.3.1 Microstructure Segmentation

The epithelial, stromal, and luminal components of each sample were segmented using the images from MR microscopy. In short, gradient echo images were used to segment luminal space, larger regions of fluid, and air bubbles; stroma was segmented using the product of fractional anisotropy (FA) and mean diffusivity (D) maps computed from DTI processing, and the remaining regions of tissue were classified as epithelium.

The MGE sequence produced four separate images each at a different echo time (TE = 16–34 ms). These images were averaged to form a single image with improved SNR, referred to as the “gradient echo image” from here on. To classify luminal space and regions of fluid (relatively long T2), a signal intensity threshold was set using the signal intensity values in the medium surrounding the samples. Additionally, a minimum intensity threshold was set to remove signal voids due to air bubbles. Thresholds were sample set dependent due to variations in image scaling.

The DTI sequence produced eight separate images: two *b* = 0 images and six *b* = 1200 s/mm^2^ images, each with a different diffusion encoding direction. Images were collectively denoised using an MP‐PCA procedure [24] (Supporting Information, Section S5) and upsampled to 20 μm isotropic resolution using linear interpolation. Following this, DTI processing [25] was performed to compute FA and *D* values for each voxel. Voxels with high diffusivity‐weighted fractional anisotropy (D*FA) were classified as stroma. Stroma has previously been shown to have a higher D and FA than epithelium [15, 26], and the product of D and FA was found to offer improved contrast between glands and stroma (with reference to aligned histology sections) compared to FA or D separately. The threshold D*FA = 1.4 × 10^−4^ mm^2^/s was empirically determined to best separate glands and stroma in benign tissue, so was used to segment stroma in all samples (benign + cancer).

Finally, all regions of tissue remaining after segmentation of lumen/fluid and stroma were classified as epithelium.

The microstructure segmentation method produced masks of epithelium, stroma, and lumen/fluid at 20 μm resolution. These masks were used to calculate ESL volume fractions in the lower‐resolution multidimensional dMRI voxels.

Alignment of gradient echo and DTI images was qualitatively assessed prior to segmentation. One sample set (2) required a small translation of the gradient echo image (200 μm) to align it with the DTI and dMRI images.

#### 2.3.2 Multidimensional dMRI Preprocessing

Multidimensional dMRI images were denoised with an MP‐PCA procedure [24] (Supporting Information, Section S6).

Following this, images from separate diffusion encoding directions were averaged to obtain direction‐averaged images. We chose to average diffusion directions since anisotropy effects are generally small for clinical voxel sizes (10–20 mm^3^) due to the heterogeneous orientation of stromal fibers [27]. Additionally, averaging over the six directions offered an SNR improvement of factor ≈2.5.

A ringing artifact was present in the *b* = 0 images due to the sharp intensity jump at the PTFE tube‐medium interface and the small acquisition matrix size (30 × 30 × 80). To reduce the impact of this artifact on the following analysis, *b* = 0 images and direction‐averaged dMRI images were downsampled to matrix size 10 × 10 × 40 (voxel size 480 × 480 × 320 μm^3^). Downsampling also enhanced SNR by a factor of ≈4.2 (N.B. neither direction averaging nor downsampling corrects for potential Rician bias in magnitude signal measurements).

Finally, direction‐averaged dMRI images were normalized by the *b* = 0 image to account for T2 heterogeneity across samples.

For each sample set, a cylindrical region was defined on the gradient echo image that enclosed all samples. Only normalized dMRI signal measurements from low resolution (480 × 480 × 320 μm^3^) voxels fully contained within the cylindrical region were included in the following analysis.

**FIGURE 1:**
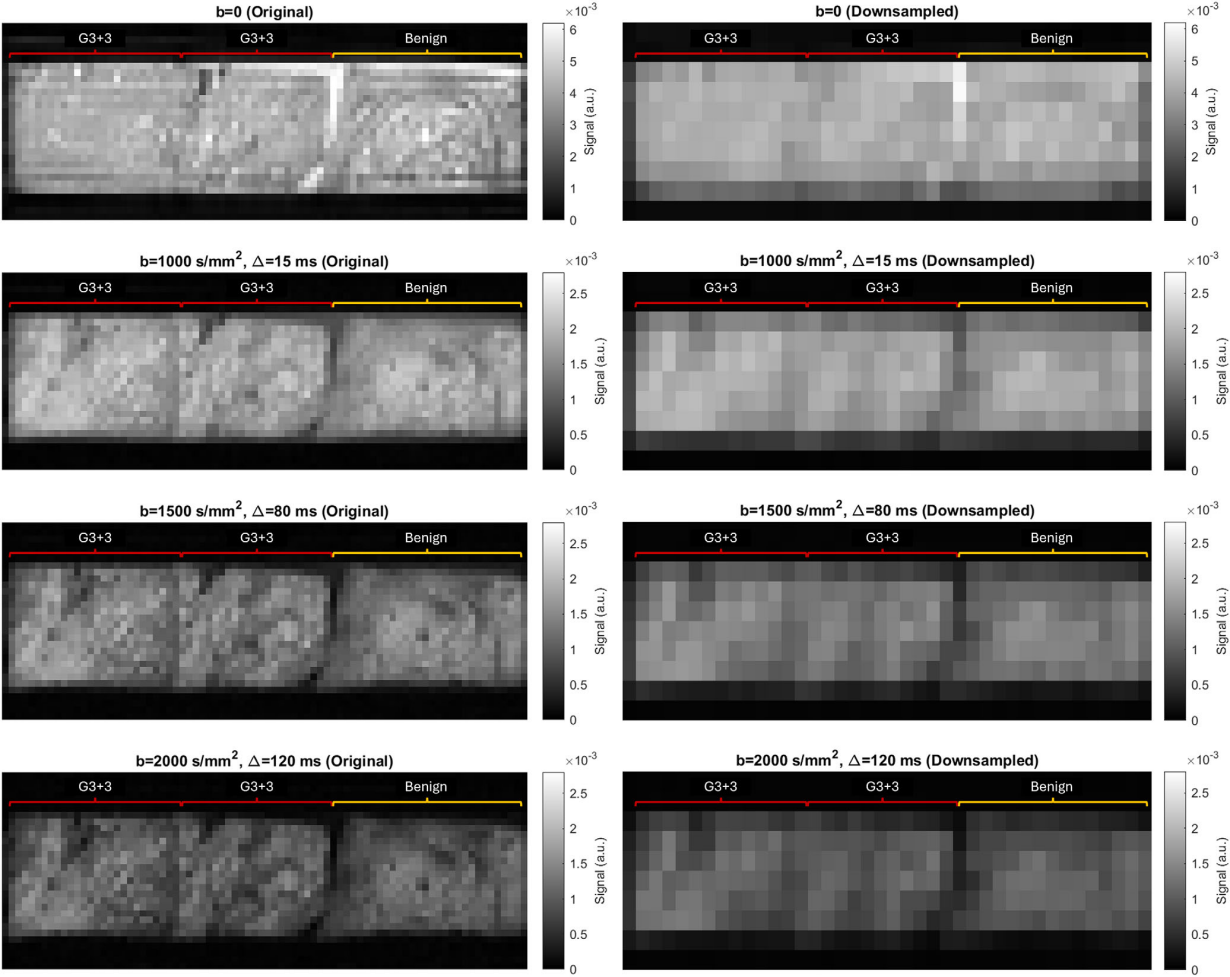
Example *b* = 0 and direction‐averaged dMRI images from sample set 1, containing samples: Gleason 3 + 3, Gleason 3 + 3, benign (left to right). The left column displays images at the acquired resolution (160 × 160 × 160 μm^3^ voxel size). At this resolution, a ringing artifact is present in the *b* = 0 image. The right column displays images at the downsampled resolution (480 × 480 × 320 μm^3^ voxel size).

Example dMRI images are displayed in Figure 1.

#### 2.3.3 Estimating Aggregate dMRI Signal Contributions From Epithelium and Stroma in Benign Tissue

**FIGURE 2:**
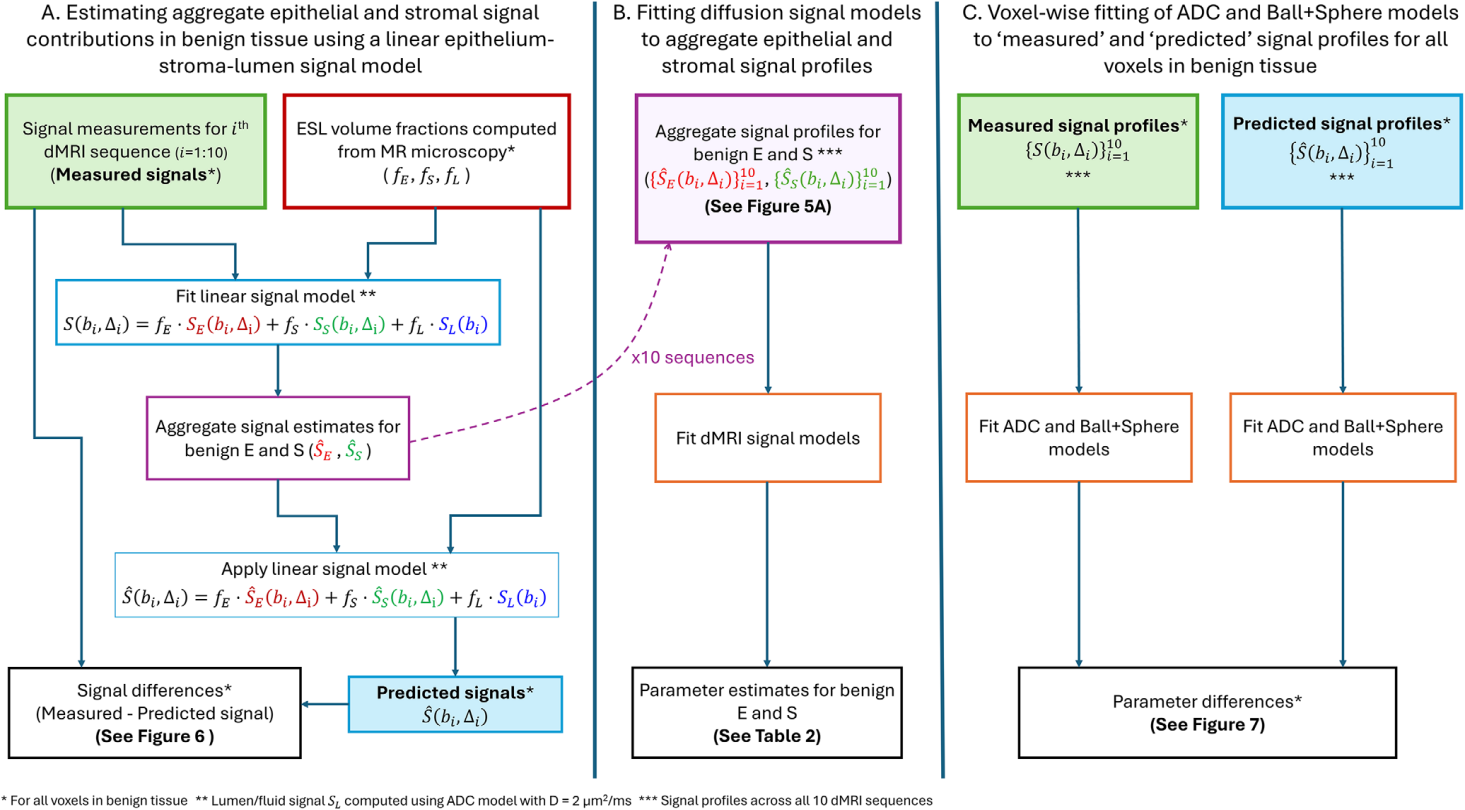
Diagram representing the analysis framework applied to benign tissue samples. (A) Method for estimating aggregate signal contributions from epithelial and stromal components in benign tissue. (B) Fitting diffusion signal models to the aggregate signal profiles estimated for benign epithelium and stroma. (C) Fitting ADC and Ball + Sphere signal models to the measured and predicted signal profiles across all voxels in benign tissue.

Figure 2 displays a flow chart representing the analysis described in Sections 2.3.3 and 2.3.4.

For each of the 10 dMRI sequences, a linear signal model was used to estimate aggregate signal contributions from epithelium and stroma in benign tissue (Figure 2 section A).

The signal from each 480 × 480 × 320 μm^3^ voxel (S) was represented as a weighted linear sum of epithelial, stromal, and luminal signal contributions ($S_{E},S_{S},S_{L}$), with weights ($f_{E},f_{S},f_{L}$) representing the volume fraction of each component within the voxel (Equation 1). Weights were calculated using the high resolution (20 μm) ESL segmentations computed from MR microscopy.

Aggregate epithelial and stromal signal contributions (${\hat{S}}_{E},{\hat{S}}_{S}$) were estimated by fitting this linear signal model to the dMRI measurement data across all benign samples. Luminal signals were fixed during fitting using the ADC model with diffusivity 2.0 μm^2^/ms, which was the diffusivity of the medium measured through DTI processing. This helped to reduce variance in epithelial and stromal signal estimates. We expect luminal fluid to have equilibrated with the surrounding medium by the time of imaging given the small size of samples and the long duration (> 24 h) of immersion in the medium [28].

(1) $$\begin{array}{cc} S\left(b_{i},\Delta_{i}\right) & =f_{E}\cdot S_{E}\left(b_{i},\Delta_{i}\right)+f_{S}\cdot S_{S}\;\left(b_{i},\Delta_{i}\right)\\ & \;+f_{L}\cdot S_{L}\left(b_{i}\right),\;i\in \left[1,2,\ldots \;,10\right] \end{array}$$

Linear model fitting was performed separately for each dMRI sequence to obtain estimates for aggregate epithelial and stromal signal contributions in benign tissue at each combination of *b*‐value and diffusion time (Δ).

Standard error and 99% confidence intervals on each aggregate signal estimate were determined through bootstrapping (*N* = 10 000). Bootstrapping was performed by randomly resampling (with replacement) the set of normalized dMRI signal measurements together with their corresponding ESL volume fractions. The linear ESL signal model was refit for each bootstrap sample. Standard errors (standard deviation) and 99% confidence intervals (0.5^th^–99.5^th^ percentiles) were calculated from the resulting distributions of signal estimates. Importantly, these measures quantify the uncertainty in the aggregate signals estimated through linear model fitting, rather than the spread of fitting residuals.

The sensitivity of microstructure segmentations and aggregate signal estimates to small changes in the chosen segmentation thresholds is investigated in the Supporting Information (Section S3).

For each sequence, the results from linear model fitting (${\hat{S}}_{E},{\hat{S}}_{S}$) were used to compute a predicted signal ($\hat{S}$) for each voxel (Equation 2). Measured and predicted signals were compared, and 95% limits of signal differences (mean ±1.96 × SD of differences) were computed. These limits quantify the extent of residual variation in measured signals not explained by the linear ESL signal model (analogous to Bland–Altman limits of agreement between measured and predicted signals).

(2) $$\begin{array}{cc} \hat{S}\left(b_{i},\Delta_{i}\right) & =f_{E}\cdot {\hat{S}}_{E}\left(b_{i},\Delta_{i}\right)+f_{S}\cdot {\hat{S}}_{S}\;\left(b_{i},\Delta_{i}\right)\\ & \;+f_{L}\cdot S_{L}\left(b_{i}\right),i\in \left[1,2,\ldots ,10\right] \end{array}$$

#### 2.3.4 Diffusion Signal Modeling in Benign Tissue

The aggregate signals estimated for benign epithelium and stroma across all 10 dMRI sequences were combined to define aggregate signal profiles for epithelium and stroma in benign tissue. Here, the term “signal profile” refers to a set of signal intensity values across the 10 dMRI sequences.

Four dMRI signal models were fit to these aggregate signal profiles (Figure 2 section B). These were: apparent diffusion coefficient (ADC, or “Ball”), diffusion kurtosis [13] (DKI), Sphere [29], and Ball + Sphere (Equations (3), (4), (5), (6)). Here, D, $D_{\text{sphere}}$, and $D_{\text{ball}}$ are diffusivities, K is the diffusion kurtosis parameter, R is the sphere radius, and $f_{\text{sphere}}$ and $f_{\text{ball}}$ are the sphere and ball‐compartment volume fractions.

(3) $$S=S_{0}\cdot e^{-\mathrm{bD}}$$

(4) $$S=S_{0}\cdot e^{-\mathrm{bD}+\frac{1}{6}Kb^{2}D^{2}}$$

(5) $$S=S_{0}\cdot \text{Sphere}\left(b,\delta ,\Delta ;R,D\right)$$

(6) $$S/S_{0}=f_{\text{sphere}}\cdot \text{Sphere}\left(b,\delta ,\Delta ;R,D_{\text{sphere}}\right)+f_{\text{ball}}\cdot e^{-bD_{\text{ball}}}$$

The Sphere function models the dMRI signal from water diffusing within impermeable spheres. An analytical expression for this function is derived by Murday and Cotts [30].

These four dMRI signal models were chosen for the following reasons: ADC is a widespread simple model that is included in current standard‐of‐care prostate imaging [7]; DKI is a common extension of the ADC model to account for non‐Gaussian diffusion in tissue; the “Sphere” model introduces sensitivity to physical restriction of diffusion in tissue and provides explicit dependence on the diffusion gradient timings (δ and Δ); and the Ball + Sphere model forms the basis of the multicompartment signal models used in VERDICT and time‐dependent dMRI.

Model fitting was performed using a least‐squares fitting procedure (“lsqcurvefit” in MATLAB). Specific fitting details (including parameter initialization and bounds) can be found in the supplied code [31]. Parameter likelihood profiles were inspected to inform choices for parameter initialization and verify that fitted parameters were associated with a well‐defined likelihood minimum.

The small‐sample corrected Akaike Information Criterion [32] (AICc) was calculated to assess the relative quality of each model for describing the aggregate signal profiles (balancing quality‐of‐fit and model complexity). Lower (more negative) AICc values indicate higher relative model quality. First‐order error propagation was used to estimate the standard error on each estimated model parameter.

Voxel‐wise diffusion model fitting was also performed (Figure 2, section C). For each voxel in benign tissue, the set of predicted signals ($\hat{S}$) across the 10 dMRI sequences were combined into a “predicted” signal profile. The ADC and Ball + Sphere models were fit to both “measured” and “predicted” signal profiles, giving two sets of parameter estimates per voxel. Parameter estimates were compared, and 95% limits of parameter differences (mean ±1.96 × SD of differences) were calculated. These limits bound the 95% spread of differences between parameter estimates obtained from measured and predicted signal profiles, and quantify the sensitivity of each model parameter to the residual variation of measured signals not explained by the linear ESL signal model.

#### 2.3.5 Diffusion Signal Modeling in Tissue Containing Cancer

The three samples containing cancer were excluded from the analysis outlined in Sections 2.3.3 and 2.3.4. and were analyzed separately.

A predicted signal profile was calculated for each voxel in cancer using the ESL volume fractions computed from MR microscopy and the aggregate signal profiles estimated for benign epithelium and stroma (Equation 2). This signal profile represents the aggregate signal predictions for a voxel of benign tissue with equivalent ESL volume fractions.

The ADC and Ball + Sphere models (Equations 3 and 6) were fit to the measured and predicted signal profiles for each voxel. Differences in parameter estimates were compared to the 95% limits of parameter differences established in benign tissue. This allowed differences in parameter estimates between benign tissue and cancer to be evaluated relative to the variability of parameter estimates in benign tissue while controlling for variation in voxel composition.

## 3 Results

### 3.1 Microstructure Segmentation

**FIGURE 3:**
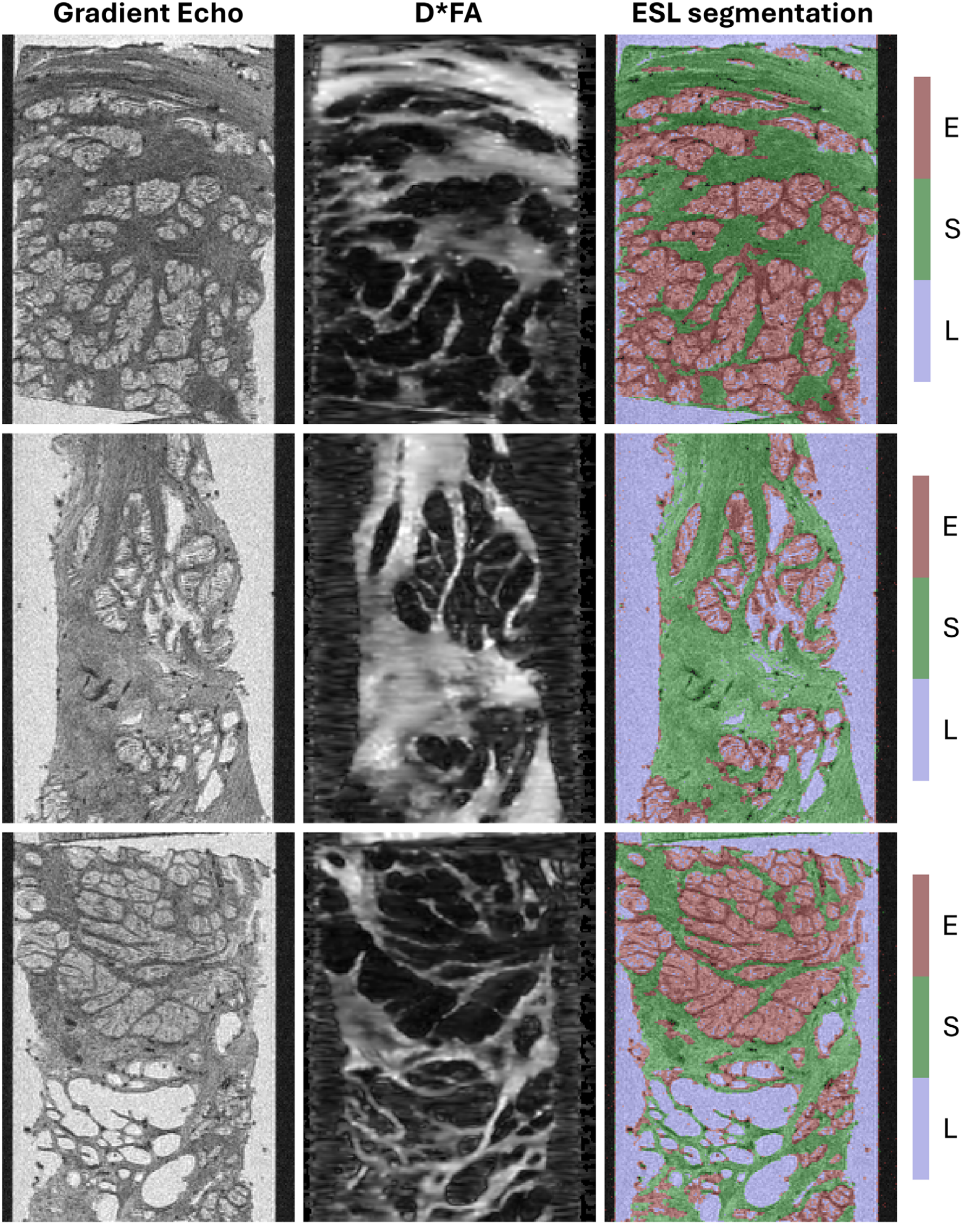
Microscopy images and microstructure segmentation results for three benign tissue samples. ESL segmentations are displayed as RGB overlays on the gradient echo image with transparency *α* = 0.2. Lumen/fluid (L) is segmented using gradient echo images, stroma (S) is segmented using the product of mean diffusivity (D) and fractional anisotropy (FA) maps from DTI processing, and the remaining regions of tissue are classified as epithelium (E).

Figure 3 displays microscopy images and microstructure segmentation results for three benign tissue samples with clear glandular structure. The gradient echo images clearly highlight regions of fluid and larger luminal spaces but offer limited contrast between epithelium and stroma, whereas the D*FA maps strongly enhance regions of stroma, but do not resolve epithelium and luminal space within glands. Combining gradient echo images and D*FA maps enables separation of ESL components.

**FIGURE 4:**
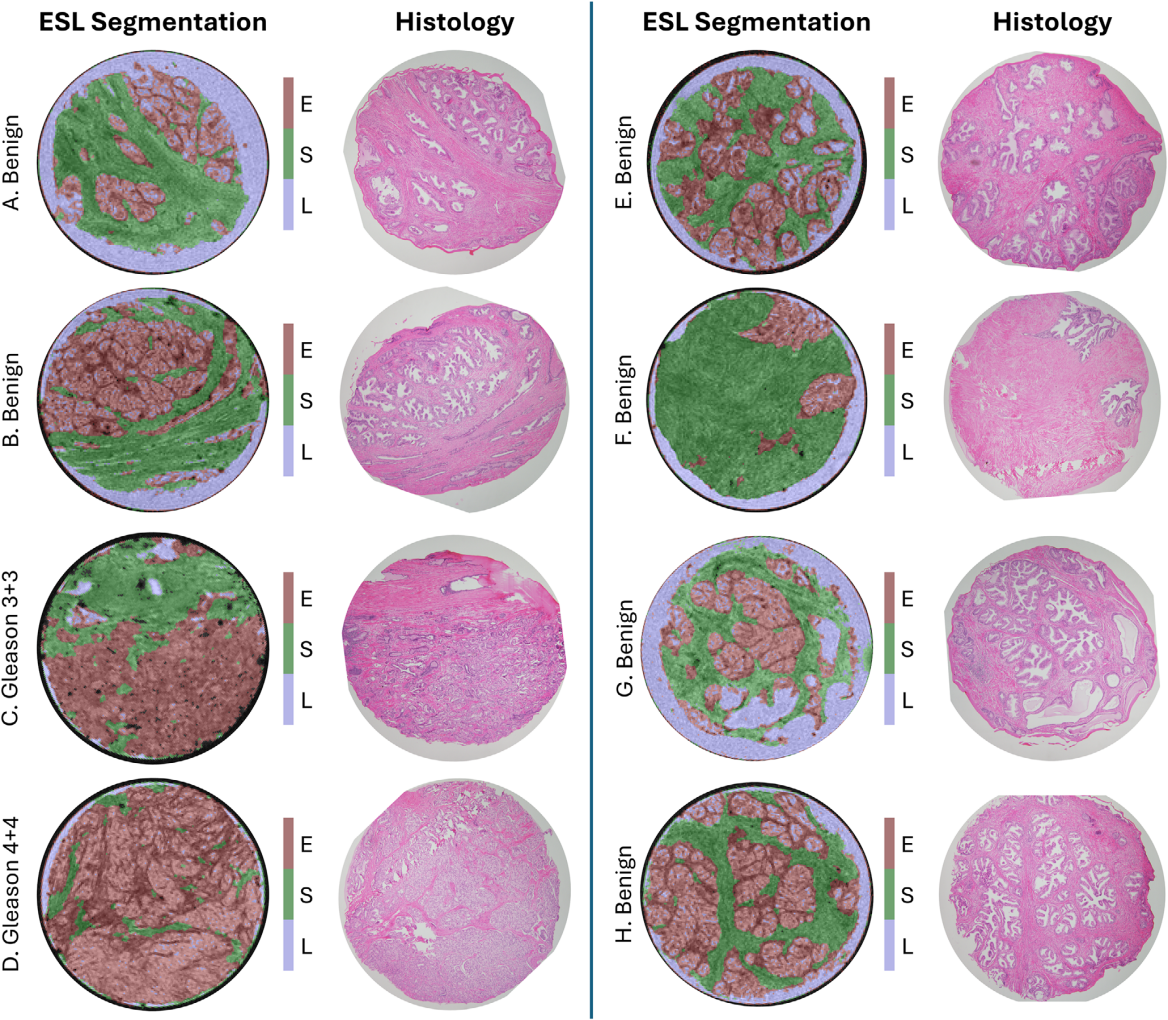
Microstructure segmentation evaluation against closest‐matching histology sections for eight samples (6× benign, 1× Gleason 3 + 3, 1× Gleason 4 + 4). ESL segmentations are displayed as RGB overlays on the gradient echo image with transparency *α* = 0.2.

Figure 4 displays example axial slices of the microstructure segmentations computed for eight samples, along with the closest‐matching histology sections. The segmentations for the benign samples show good agreement with the glandular structure seen in histology; however, some of the smaller luminal spaces within glands appear larger in histology than in gradient echo images.

The sample containing Gleason 3 + 3 cancer includes an extended region of tissue classified almost entirely as epithelium. Within this region, histology images display a dense arrangement of small glands with minimal luminal space and surrounding stroma, indicating that the computed segmentations are largely consistent with the microstructure of the tissue. Histology images for the sample containing Gleason 4 + 4 cancer show extended regions of dense epithelium with no clear glandular structure; these regions are correctly classified as epithelium by the segmentation method.

Overall, the segmentations computed from MR microscopy show good visual agreement with the microstructure observed in histology.

### 3.2 Estimating Aggregate dMRI Signal Contributions From Epithelium and Stroma in Benign Tissue

In total, 3806 low‐resolution (480 × 480 × 320 μm^3^) voxels from the 14 benign samples were included in the analysis of dMRI signals from benign tissue. Across all voxels, the average volume fractions of epithelium, stroma, lumen/fluid, and unclassified space (below gradient echo minimum signal threshold) computed from microstructure segmentation were 27.8%, 57.8%, 14.0%, and 0.4%, respectively. The distributions of epithelial, stromal, and luminal/fluid volume fractions across all benign voxels are displayed in the Supporting Information (Section S4).

**FIGURE 5:**
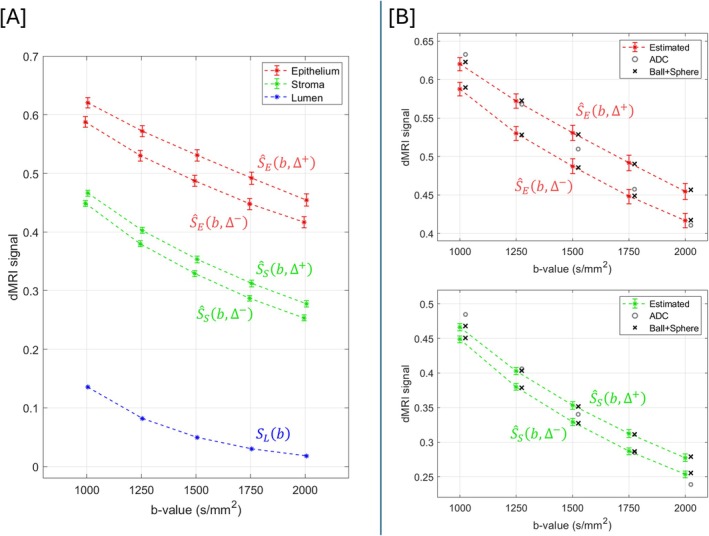
(A) Aggregate signal estimates for epithelium and stroma in benign tissue. For both components, there are two aggregate signal estimates per *b*‐value: one for the sequence with shorter diffusion time (Δ^−^) and one for the sequence with longer diffusion time (Δ^+^). (B) Aggregate signal profiles for epithelium and stroma in benign tissue with signal values following ADC and Ball + Sphere model fitting shown with additional markers.

Figure 5A displays the aggregate signal profiles estimated for epithelium and stroma in benign tissue. For all 10 dMRI sequences, the aggregate signal estimates returned from linear model fitting were higher for epithelium than for stroma. The contrast between estimated epithelial and stromal signals is maximal at *b* = 1750 s/mm^2^, Δ = 100 ms, although the differences in this contrast between sequences are small. At each *b*‐value, signal estimates for both epithelium and stroma were higher for the sequence with longer diffusion time (Δ^+^); however, this difference was more pronounced for epithelium. The confidence intervals determined through bootstrapping were small for all sequences, indicating that the number of low‐resolution dMRI voxels and range of epithelial and stromal volume fractions were sufficient for reliable linear model fitting.

**FIGURE 6:**
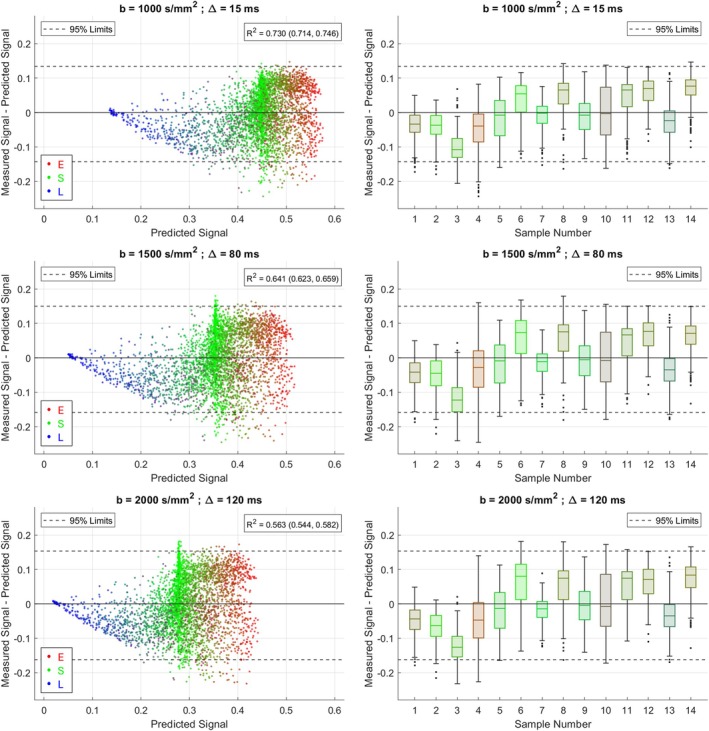
Comparison of measured and predicted signals across all voxels in benign tissue for three example sequences. In the lefthand subplots, each marker corresponds to an individual voxel and is colored with RGB values that reflect the volume fractions of epithelium, stroma, and lumen within the voxel, respectively. In the righthand subplots, signal differences are separated by sample. Boxplots are colored with RGB values corresponding to the average epithelial, stromal, and luminal volume fractions across all voxels within the sample. In each plot, the 95% limits of signal differences (across all voxels) are displayed with dashed horizontal lines.

Figure 6 displays the differences between measured and predicted signals in benign tissue for three example sequences. In each case, the vertical spread of datapoints reflects residual variation in measured signals not explained by the linear ESL signal model. The *R* ^2^ values obtained from linear model fitting range from 0.563 to 0.737; the lower values come from the higher *b*‐value sequences, indicating that residual signal variation is proportionally greater for these sequences.

Figure 6 also displays the signal differences separated by sample. The boxplots for individual samples are generally not centred around zero and the relative positions of boxplots are similar for each sequence.

### 3.3 Diffusion Signal Modeling in Benign Tissue

Table 2 displays the results from fitting the four dMRI signal models to the aggregate signal profiles estimated for benign epithelium and stroma. Within the ADC model, a lower diffusivity was estimated for epithelium (0.433 ± 0.0019 μm^2^/ms) compared to stroma (0.708 ± 0.0014 μm^2^/ms) (parameter estimate ± standard error); however, the AICc values for both tissue components were relatively high, indicating that the ADC model does not describe the signal profiles well.

**TABLE 2:** Results from model fitting to aggregate signal profiles estimates for epithelium and stroma in benign tissue.

Component	Model name	Estimated parameters	AICc
Epithelium	ADC (‘Ball’)	**S**_**0**_ = 0.975 (0.0013); **D** = 0.433 (0.0019) μm^2^/ms	−73.4
Epithelium	DKI	**S**_**0**_ = 0.999 (0.0003); **D** = 0.584 (0.0071) μm^2^/ms; **K** = 1.52 (0.043)	−77.3
Epithelium	Sphere	**S**_**0**_ = 0.995 (0.0010); **D** = 0.564 (0.0051) μm^2^/ms; ***R*** = 23.5 (0.58) μm	−99.3
Epithelium	Ball + Sphere	**S**_**0**_ = 1.000 (0.0003); **f**_**sphere**_ = 0.278 (0.024); **D**_**sphere**_ = 0.641 (0.131) μm^2^/ms; ***R*** = 6.42 (0.45) μm; **D**_**ball**_ = 0.611 (0.032) μm^2^/ms	−118.2
Stroma	ADC (‘Ball’)	**S**_**0**_ = 0.983 (0.0005); **D** = 0.708 (0.0014) μm^2^/ms	−78.4
Stroma	DKI	**S**_**0**_ = 1.000 (0.0001); **D** = 0.899 (0.0055) μm^2^/ms; **K** = 0.885 (0.016)	−89.8
Stroma	Sphere	**S**_**0**_ = 0.994 (0.0004); **D** = 0.847 (0.0040) μm^2^/ms; ***R*** = 36.5 (0.74) μm	−95.6
Stroma	Ball + Sphere	**S**_**0**_ = 1.000 (0.0001); **f**_**sphere**_ = 0.175 (0.0088); **D**_**sphere**_ = 0.532 (0.072) μm^2^/ms; ***R*** = 6.38 (0.42) μm; **D**_**ball**_ = 0.943 (0.015) μm^2^/ms	−124.0

*Note*: See Equations ((3), (4), (5), (6)) for the mathematical representations of each model. Lower (more negative) AICc values indicate higher relative model quality (balancing quality‐of‐fit and model complexity). The standard error on each estimated model parameter determined through first order uncertainty propagation is displayed in brackets.

The Ball + Sphere model provided the best fit (lowest AICc value) to both signal profiles. Differences (beyond the estimated parameter error) between epithelium and stroma were found for the sphere fraction and ball‐compartment diffusivity parameters; a higher sphere fraction (0.278 ± 0.024 compared to 0.175 ± 0.009)and lower ball‐compartment diffusivity (0.611 ± 0.032 compared to 0.943 ± 0.015 μm^2^/ms) were estimated for epithelium. The sphere radius and sphere‐compartment diffusivity estimates were similar for both tissue components.

Compared to the Ball + Sphere model, the DKI and Sphere models provided relatively poor fits to the signal profiles for both components so are not discussed further. The ADC model is included in further analysis as a reference for evaluating Ball + Sphere modeling results.

Figure 5B visualizes the quality‐of‐fit provided by the ADC and Ball + Sphere models. The Ball + Sphere model describes the aggregate signal profiles for both epithelium and stroma closely; however, the ADC model provides a poor fit as it cannot describe the dependence of estimated signals on diffusion time (Δ).

**FIGURE 7:**
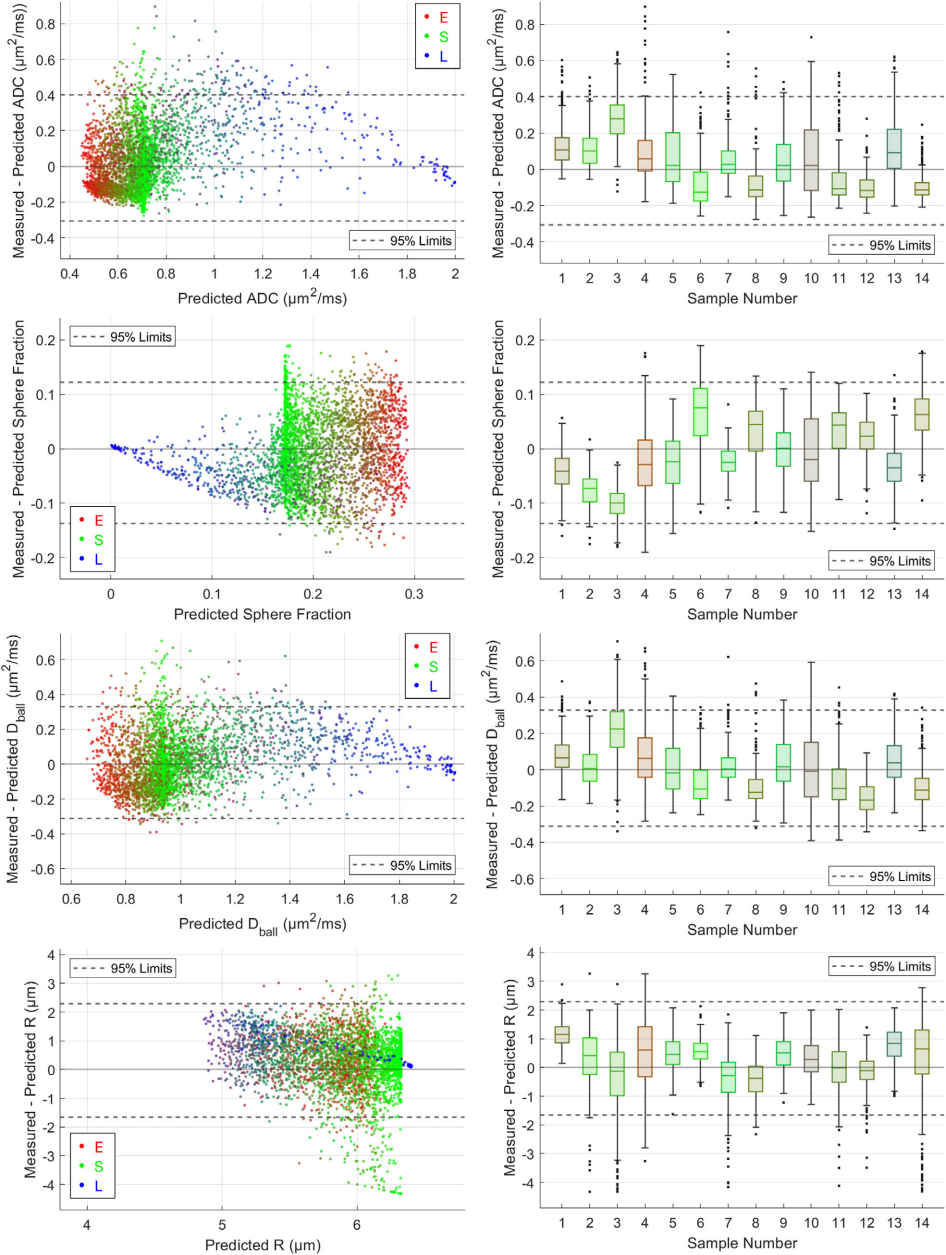
Comparison of ADC and Ball + Sphere model parameter estimates obtained from fitting to measured and predicted signal profiles across all voxels in benign tissue. In the lefthand subplots, each marker corresponds to an individual voxel and is colored with RGB values corresponding to the volume fractions of epithelium, stroma, and lumen within the voxel, respectively. In the righthand subplots, parameter differences are separated by sample. Boxplots are colored with RGB values corresponding to the average epithelial, stromal, and luminal volume fractions across all voxels within the sample. In each plot, the 95% limits of parameter differences (across all voxels) are displayed with dashed horizontal lines.

Figure 7 displays the differences between model parameters estimated from fitting to the measured and predicted signal profiles across all voxels in benign tissue. As a proportion of the parameter value estimated for benign epithelium, the ADC 95% limits are broadest; however, the sphere fraction limits are wider relative to the range of predicted parameter values.

The narrow vertical band of green datapoints in the sphere fraction plot mostly originates from a sample composed almost entirely of stroma (sample number 6). Measured signals from this sample were higher than the aggregate signals estimated for benign stroma, leading to abnormally high sphere fraction estimates; however, the raised signals from this sample had a less pronounced impact on ADC and the other Ball + Sphere model parameters.

### 3.4 Diffusion Signal Modeling in Tissue Containing Cancer

**FIGURE 8:**
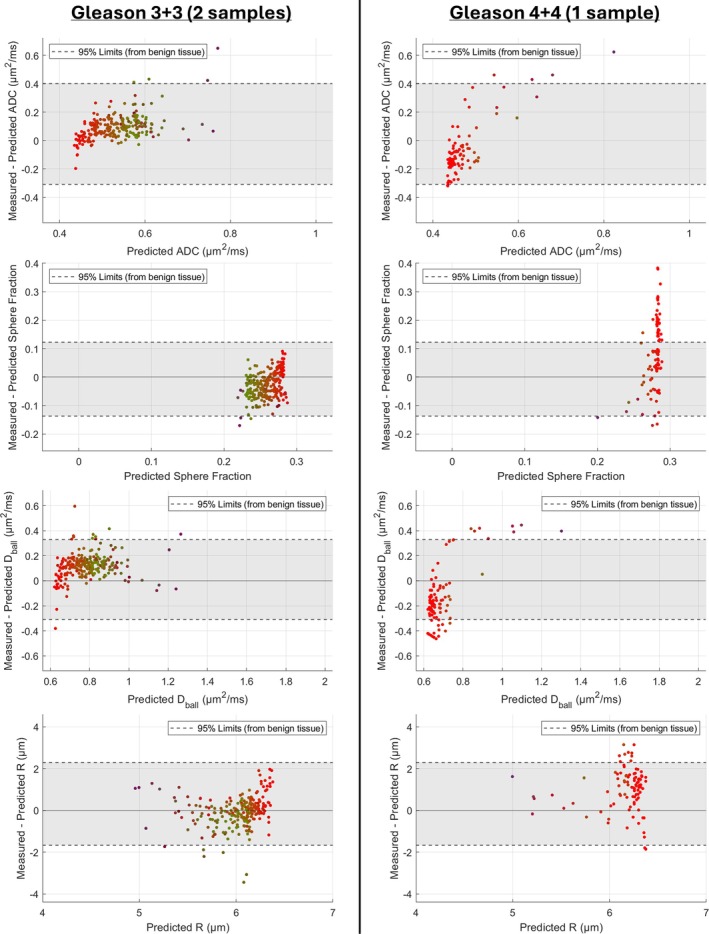
Comparison of ADC and Ball + Sphere model parameter estimates obtained from fitting to measured and predicted signal profiles across voxels in samples containing cancer. The lefthand subplots display the combined results for the two samples containing Gleason 3 + 3 cancer. The righthand subplots display the results for the one sample containing Gleason 4 + 4 cancer. In all subplots, each marker corresponds to an individual voxel and is colored with RGB values corresponding to the volume fractions of epithelium, stroma, and lumen within the voxel, respectively. The 95% limits of parameter differences established in benign tissue are displayed with dashed horizontal lines, and the region within these limits is shaded gray.

Figure 8 displays the differences between ADC and Ball + Sphere parameter estimates obtained from fitting to the measured and predicted signal profiles in samples containing cancer.

For the two samples containing Gleason 3 + 3 cancer, the majority of parameter differences fall within the 95% limits established in benign tissue, so are smaller than the residual variation of parameter estimates seen in benign tissue.

For the sample containing Gleason 4 + 4 cancer, larger parameter differences are observed. Most notably, an increase to sphere fraction estimates is seen, with many differences exceeding the 95% limits established in benign tissue. However, the vertical spread of data points indicates that there is still considerable heterogeneity in sphere fraction estimates across the sample. The differences seen for the ball‐compartment diffusivity and sphere radius parameters are mostly contained within the 95% limits, though larger differences are seen for a small number of voxels. An overall reduction in ADC estimates is observed; however, almost all differences fall within the 95% limits established in benign tissue.

## 4 Discussion

In this work, we performed paired MR microscopy and multidimensional dMRI to segment the epithelial, stromal, and luminal components of prostate tissue samples, and characterize the diffusion properties of epithelium and stroma in both benign tissue and cancer.

The microstructure segmentation method enabled the ESL volume fractions of all voxels across all samples to be efficiently resolved, while avoiding the registration challenges associated with direct MRI‐histology comparisons. Manual segmentation of this many voxels would be impractical. Further, segmentation and analysis of entire samples removes the potential for sampling bias associated with manual ROI‐based approaches, and enables better assessment of the variation in diffusion properties both within and across samples.

Overall, the segmentations from MR microscopy showed good visual agreement with the microstructure seen in histology. However, gradient echo images generally provided limited contrast between epithelium and small luminal spaces within glands, so segmentations of the epithelium and lumen/fluid components were sensitive to the chosen gradient echo signal threshold. Luminal spaces generally appeared larger in histology than in gradient echo images, though this can partially be attributed to epithelial shrinkage during histological processing [33]. The lack of gradient echo signal contrast between epithelium and lumen could be due to partial volume effects, or could indicate a difference in T2 between luminal fluid and the surrounding medium, possibly arising from luminal secretions or proteins that do not readily diffuse out of the tissue. At the gradient echo signal threshold used in this work, it is likely that epithelial segmentations include small luminal spaces. As a result, the aggregate epithelial signals estimated in this work may underestimate the dMRI signal contributions from pure epithelium.

The results from T2 measurement show that epithelium and stroma have distinct relaxation properties. Consequently, voxel‐wise normalization of dMRI signal measurements by the *b* = 0 image does not fully remove the influence of relaxation. However, we expect the resulting impact on aggregate signal estimates to be small (Supporting Information, Section S2).

The aggregate signal profiles estimated for benign epithelium and stroma were both best described by the Ball + Sphere model. A previous study [34] also showed that the Ball + Sphere model provides an improved description of dMRI signal from fixed prostate tissue compared to ADC, though this study performed modeling on large heterogeneous voxels, rather than considering epithelial and stromal components separately.

The diffusivities reported from fitting the ADC model to the aggregate signal profiles estimated for epithelium and stroma are consistent with the results from a previous MR microscopy study on fixed prostate tissue [28]. This supports the physical plausibility of the aggregate signal profiles estimated for both components.

Within the Ball + Sphere model, a higher sphere fraction and lower ball‐compartment diffusivity were found for epithelium, indicating a higher volume fraction of restricted water and lower mobility for unrestricted water. Epithelium is widely recognized to consist of densely arranged cells with tight intercellular junctions and minimal extracellular space, whereas stroma contains a lower density of cells with an extensive extracellular matrix. The differences in sphere fraction and ball‐compartment diffusivity parameter estimates between epithelium and stroma are consistent with these microstructural differences, though the sphere fraction value estimated for epithelium was perhaps lower than anticipated [35]. This could reflect permeability of epithelial cell membranes, or may be the result of small luminal spaces being included in epithelial segmentations.

Despite the clear differences between aggregate epithelial and stromal signals, considerable variation in measured signals was observed that was not explained by the linear ESL signal model. The differences between measured and predicted signals showed noticeable inter‐sample variation, with the sign and scale of differences largely consistent between sequences; this is evidence that the observed residual variation is not random, but rather reflects systematic differences in the dMRI signals measured across samples.

Some residual variation in measured signals could be attributed to segmentation inaccuracies. However, the observed differences between measured and predicted signals were often comparable in magnitude to the differences between aggregate epithelial and stromal signal estimates. Attributing residual variation of this scale to segmentation inaccuracies alone would imply complete mis‐segmentation of epithelium as stroma, or vice versa. Although the segmentations were not perfect, we do not broadly see this level of inaccuracy when comparing segmentations to histology, so the residual variation of measured signals is more likely to reflect genuine heterogeneity in the microscopic diffusion properties of tissue components across samples.

Residual variation in measured signals may also arise from differing degrees of stromal fiber alignment within low‐resolution dMRI voxels. However, simulation results suggest that signal variability due to this effect is small compared to the observed residual variation in measured signals (Supporting Information, Section S7).

Notably, good agreement between measured and predicted signals was observed for voxels with high lumen/fluid content (cluster of dark blue markers). This indicates that the fixed medium diffusivity was appropriate for these voxels, and there was minimal Rician noise bias in the high‐*b*‐value signal measurements.

The residual variation in measured signals is reflected in the differences between ADC and Ball + Sphere model parameters estimated from measured and predicted signal profiles. While model fitting to aggregate epithelial and stromal signal profiles showed distinct differences that align with known epithelial and stromal microstructure, the parameters estimated from voxel‐wise model fitting showed considerable variability beyond that predicted by the three‐component ESL tissue description. If model parameters are intended to be used as biomarkers for disease, it is important to be aware of the potential variation in parameter estimates across benign tissue, as this defines the baseline variability against which pathological changes must be detected.

The three‐component ESL tissue description is central to the analysis included in this work; however, the results presented for benign tissue suggest that the underlying prostate tissue microstructure is markedly more complex, so care should be taken when interpreting the results from such simplified tissue models.

The ADC and Ball + Sphere parameter estimates in the two samples containing Gleason 3 + 3 cancer were largely consistent with the parameter values found for benign epithelium and stroma. This suggests that the diffusion properties of epithelium in Gleason pattern 3 and benign epithelium are similar. However, the diffusion properties of epithelium in the Gleason 4 + 4 sample did appear to differ, most strongly reflected through an increase to sphere fraction estimates. The raised sphere fraction estimates signify an increase to the volume fraction of restricted water within the tissue; this is consistent with an increase to epithelial cell density and possibly stems from invasive growth of epithelium into small luminal spaces that would not have been resolved through MR microscopy.

The change in ADC estimates between Gleason 3 + 3 and Gleason 4 + 4 cancer was less pronounced, indicating that the ADC parameter is less sensitive to the microstructural changes between Gleason pattern 3 and 4 epithelium.

The VERDICT imaging technique [4] utilizes a signal model that includes “Ball” and “Sphere” compartments, and there is emerging evidence that the intracellular volume fraction (sphere fraction) parameter estimated through this technique could offer improved distinction between clinically significant cancer (Gleason ≥ 3 + 4) and nonsignificant lesions (Gleason ≤ 3 + 3) than ADC [36, 37]. The results from this work broadly support these previous findings; however, as the number of samples containing cancer is small, grade‐specific observations are preliminary and require cautious interpretation.

In this work, we have presented a framework for characterizing the microscopic diffusion properties of prostate tissue using paired MR microscopy and multidimensional dMRI. The framework has a number of limitations that should be acknowledged.

The detail of microstructure “ground truth” maps obtainable through MR microscopy is limited by the contrast and resolution of the chosen microscopy sequences. While the microscopy sequences included in this work generally enabled the main prostate tissue components (ESL) to be distinguished, many of the histological features used in Gleason grading have length scales below the achievable resolution of MR microscopy. Therefore, future studies should use MR microscopy in combination with histology to draw on the benefits of both techniques.

The segmentation process employed in this work was simplistic, based on fixed thresholds within each sample set. This led to ambiguous and threshold‐sensitive segmentation of epithelium and luminal space within glands. Future studies could adopt more advanced segmentation methods that incorporate prior knowledge of glandular structure. This may improve segmentation performance and aid the generalizability of results.

The prostate tissue samples included in this work were fixed with formalin to preserve microstructure during imaging and histological processing. Formalin‐fixation is known to cause some tissue shrinkage [38] and reduces the mobility of water in the tissue due to protein cross‐linking [39]. Also, there is no vascular perfusion in fixed tissue. Therefore, the dMRI signal measurements and model parameter estimates found in this work may not be directly representative of in vivo tissue. However, since fixation preserves microstructure, we expect the broader conclusions to translate to unfixed tissue.

## 5 Conclusion

Direction‐averaged diffusion in fixed prostate epithelium and stroma is well described by the Ball + Sphere model; the parameters estimated from aggregate epithelial and stromal signal profiles reflect the known microstructural differences between components. However, the residual variation in signal measurements and model parameter estimates across the benign samples indicates that the three‐component epithelium‐stroma‐lumen tissue description is too simplistic to capture the full microstructural heterogeneity of benign prostate tissue.

The diffusion properties of epithelium in benign tissue and Gleason 3 + 3 cancer appear to be similar; however, a marked increase in the volume fraction of restricted water was found for epithelium in Gleason 4 + 4 cancer. These preliminary findings indicate distinct diffusion behavior in Gleason pattern 3 and Gleason pattern 4 epithelium, so support the potential for non‐invasive prostate cancer grading using quantitative dMRI. Further imaging of samples containing cancer is required to generalize these conclusions.

## Funding

This work is supported by the Engineering and Physical Sciences Research Council funded UCL Centre for Doctoral Training in Intelligent, Integrated Imaging in Healthcare (i4health) (EP/S021930/1).

## Supporting information


**Data S1:** mrm70344‐sup‐0001‐Supinfo.docx.

## Data Availability

All imaging data can be made available upon request to the corresponding author. The MATLAB code used for image processing and data analysis is available on GitHub [31].
