# Supplementary material for: Characterizing the Diffusion Properties of Prostate Tissue Using Paired MR Microscopy and Multidimensional Diffusion MRI
Source: Magn Reson Med. 2026 Mar 24;96(1):349–65. doi: 10.1002/mrm.70344 (PMC13156426; doi:10.1002/mrm.70344)
Supplement: Supplementary file 1 — Data S1: mrm70344‐sup‐0001‐Supinfo.docx. [file MRM-96-349-s001.docx]

# Supplementary Materials

## Sample details

| Sample set | No. samples | Sample location | Pathologist report  (top-bottom) |
| --- | --- | --- | --- |
| 1 (UQ4, Specimen 1) | 3 | UQ4a - Slice 3, right posterior region  UQ4b – Slice 4, left posterior region  UQ4c – Slice 4, right posterior region | UQ4a – Gleason 3+3  UQ4b – Gleason 3+3  UQ4c – Benign |
| 2 (UQ5, Specimen 2) | 3 | UQ5a – Slice 3, posterior region  UQ5b – Slice 4, left anterior region  UQ5c – Slice 4, right anterior region | UQ5a – Benign  UQ5b – Benign  UQ5c – Benign |
| 3 (UQ6, Specimen 2) | 3 | UQ6a – Slice 5, left posterior region  UQ6b – Slice 5, right posterior region  UQ6c – Slice 4, left mid-gland region | UQ6a – Benign  UQ6b – Benign  UQ6c – Gleason 4+4 |
| 4 (UQ7, Specimen 3) | 3 | UQ7a – Slice 2, right anterior region  UQ7b – Slice 2, left posterior region  UQ7c – Slice 2, right posterior region | UQ7a – Benign  UQ7b – Benign  UQ7c – Benign |
| 5 (UQ8, Specimen 3) | 3 | UQ8a – Slice 2, right posterior region  UQ8b – Slice 3, right anterior region  UQ8c – Slice 3, right anterior region | UQ8a – Benign  UQ8b – Benign  UQ8c – Benign |
| 6 (UQ9, Specimen 3) | 2 | UQ9a – Slice 3, right posterior region  UQ9b – Slice 3, left posterior region | UQ9a – Benign  UQ9b – Benign |

Table S1: prostate tissue sample details

## T2 distributions in samples

The T2 values across each set of samples was calculated using images from the MSME sequence (Table 1). Figures S1-S6 display histograms showing T2 values from within rectangular regions that enclosed all samples within the NMR tube (displayed in red).

### Sample set 1 (UQ4)

Gleason 3+3, Gleason 3+3, Benign (top-bottom)


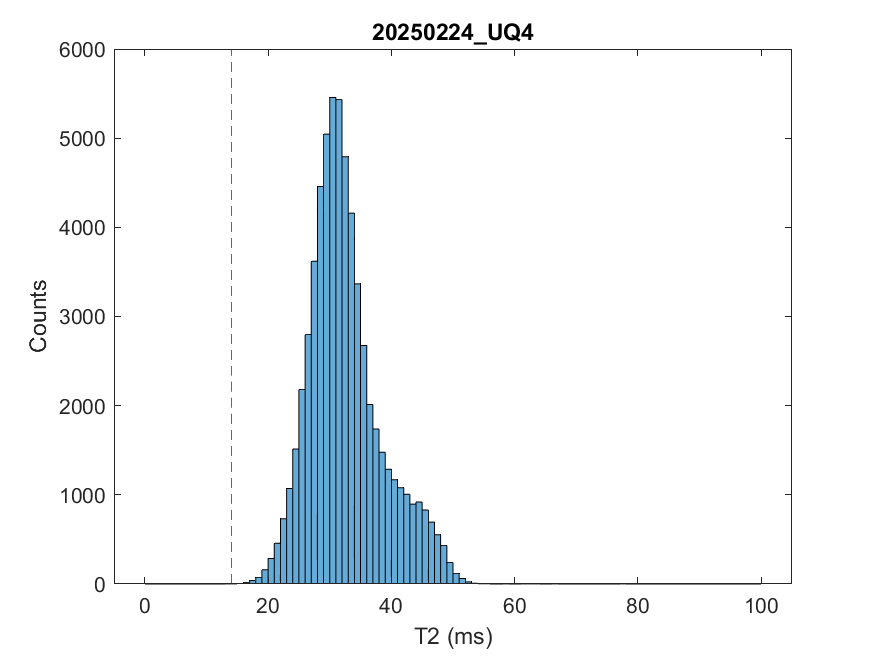

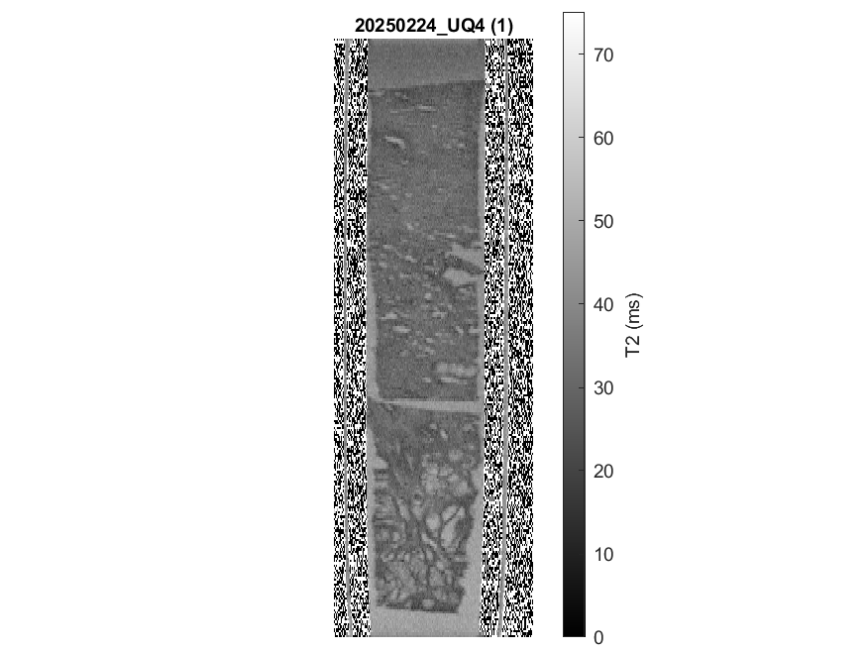

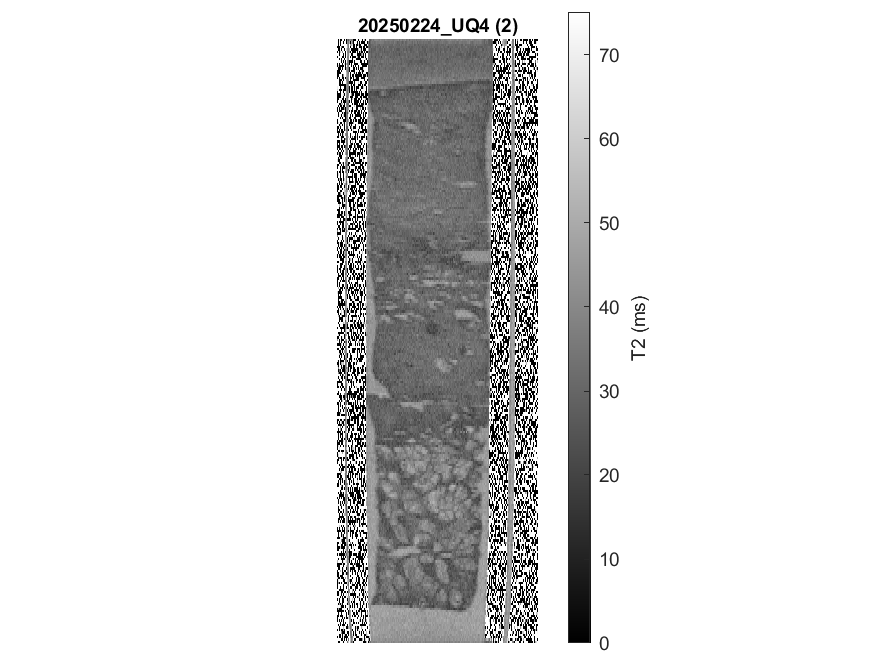


Figure S1: Sample UQ4 T2 distribution

### Sample set 2 (UQ5)

Benign, Benign, Benign (top-bottom)


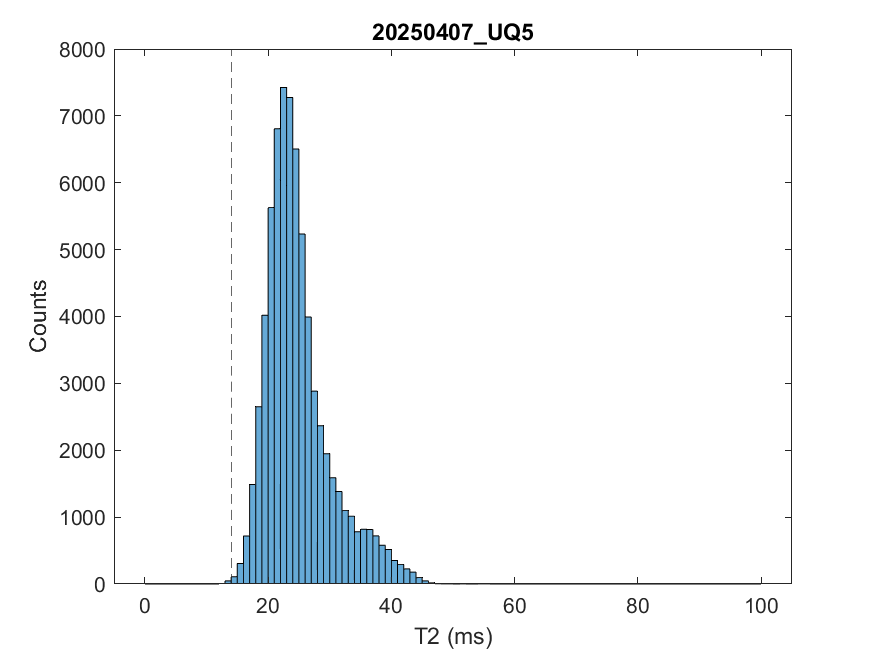

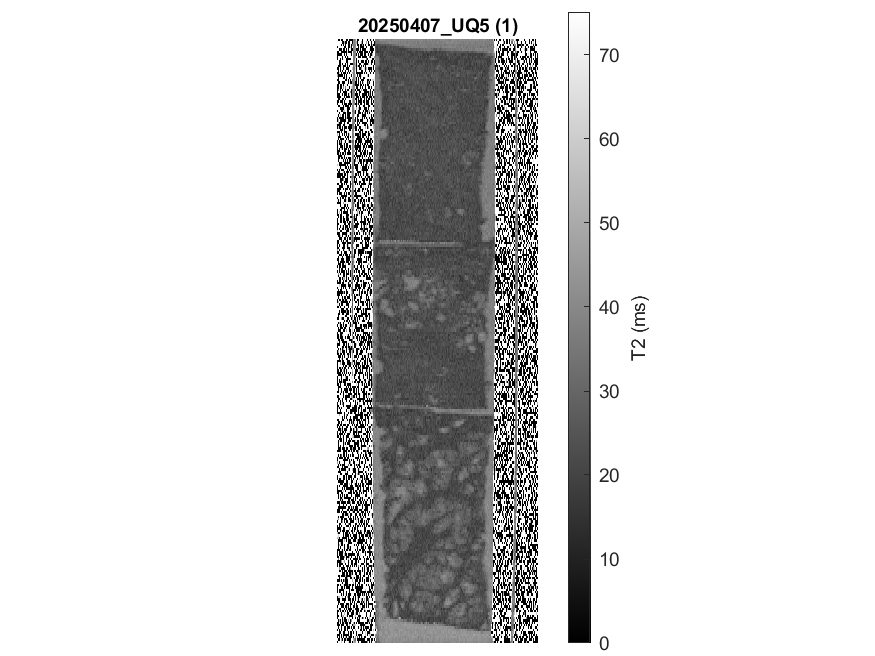

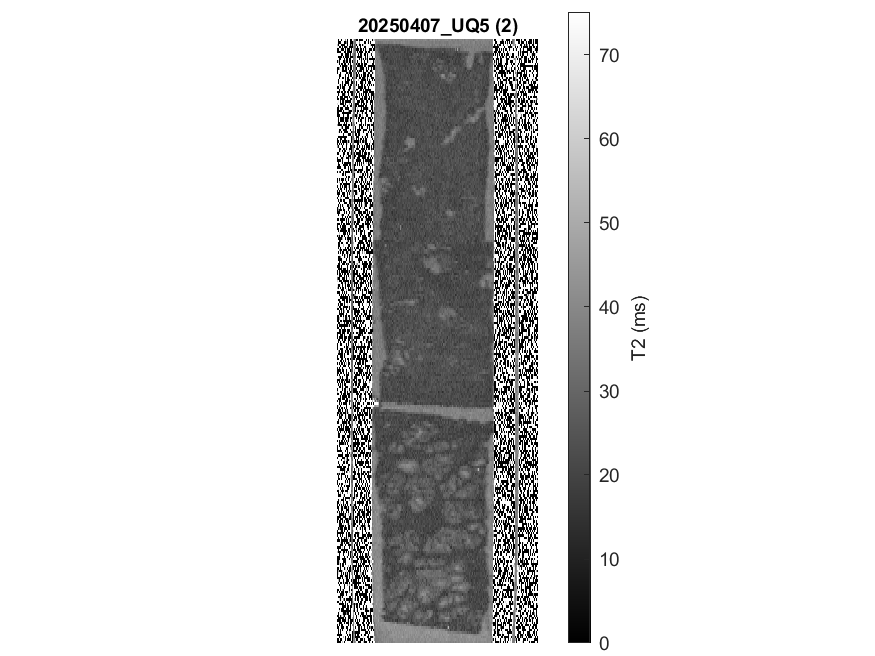


Figure S2: Sample UQ5 T2 distribution

### Sample set 3 (UQ6)

Benign, Benign, Gleason 4+4 (top-bottom)


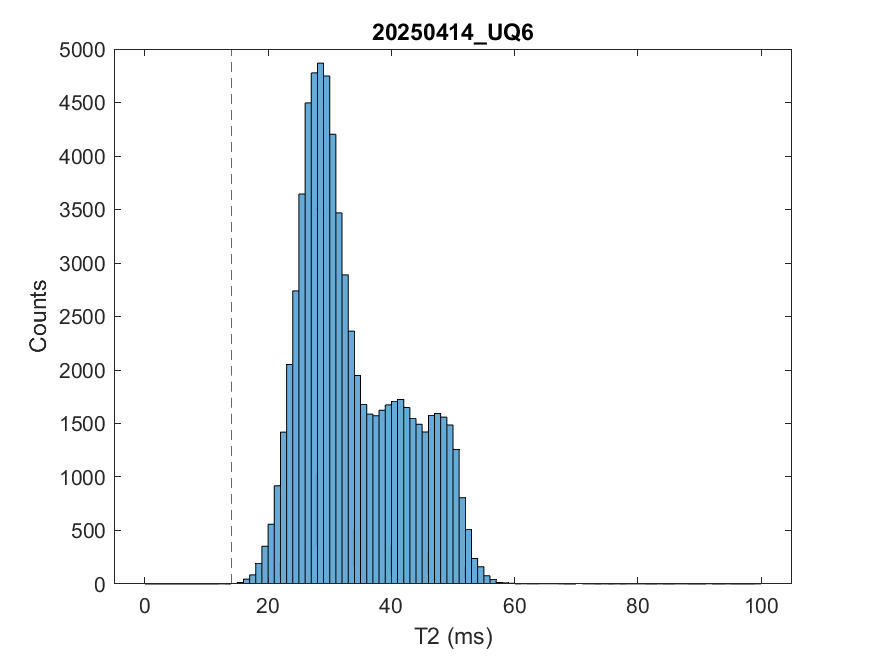

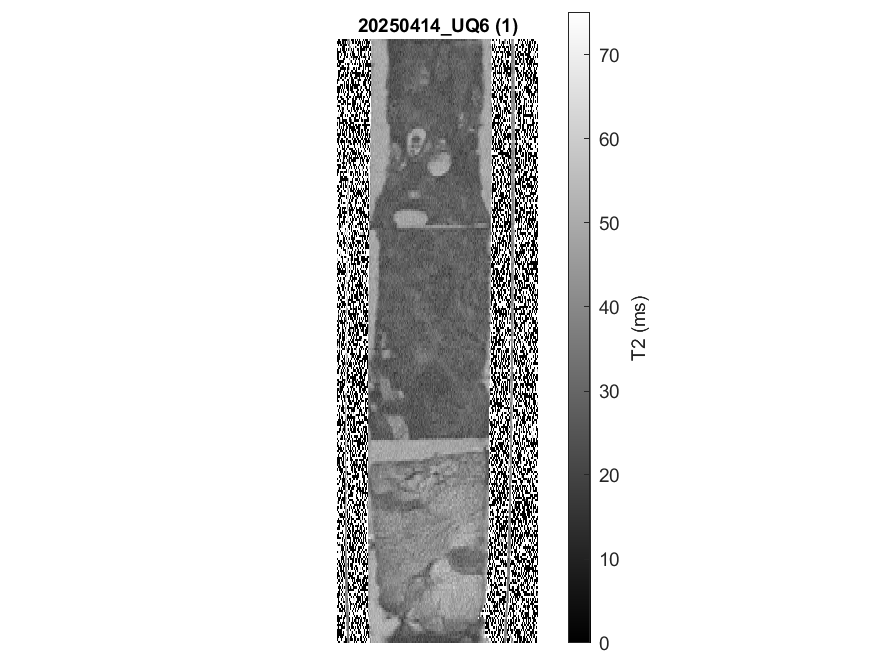

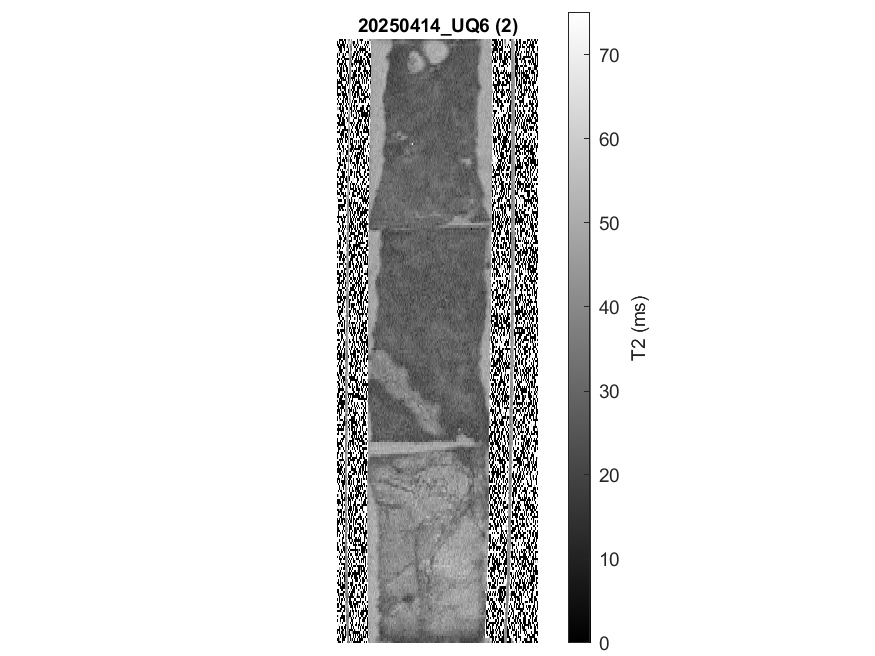


Figure S3: Sample UQ6 T2 distribution

### Sample set 4 (UQ7)

Benign, Benign, Benign (top-bottom)


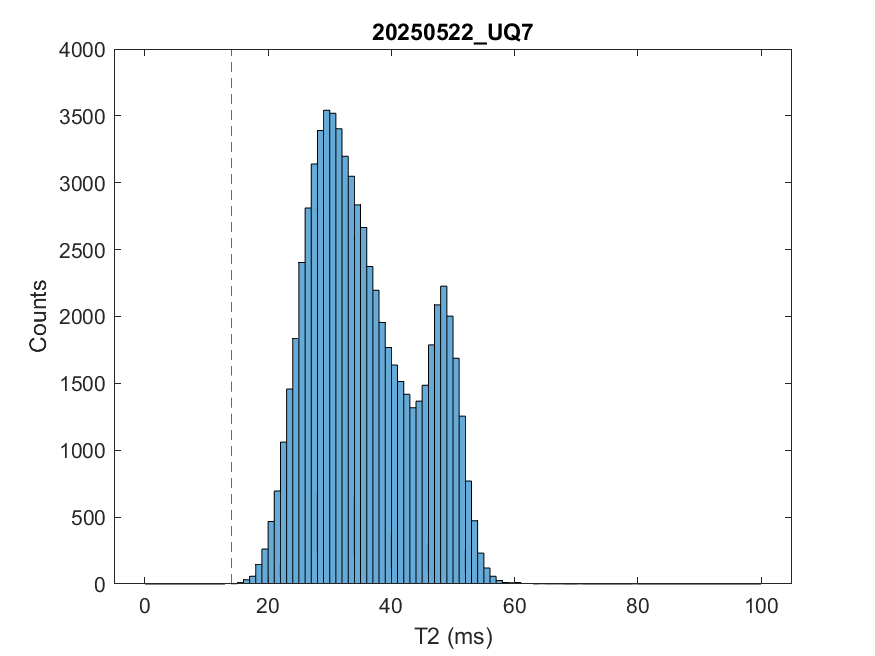

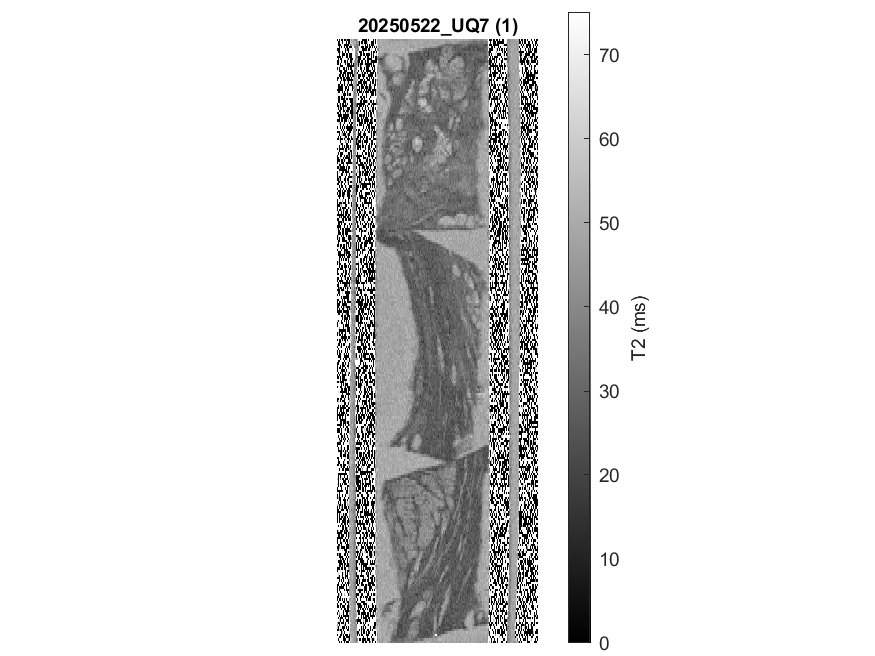

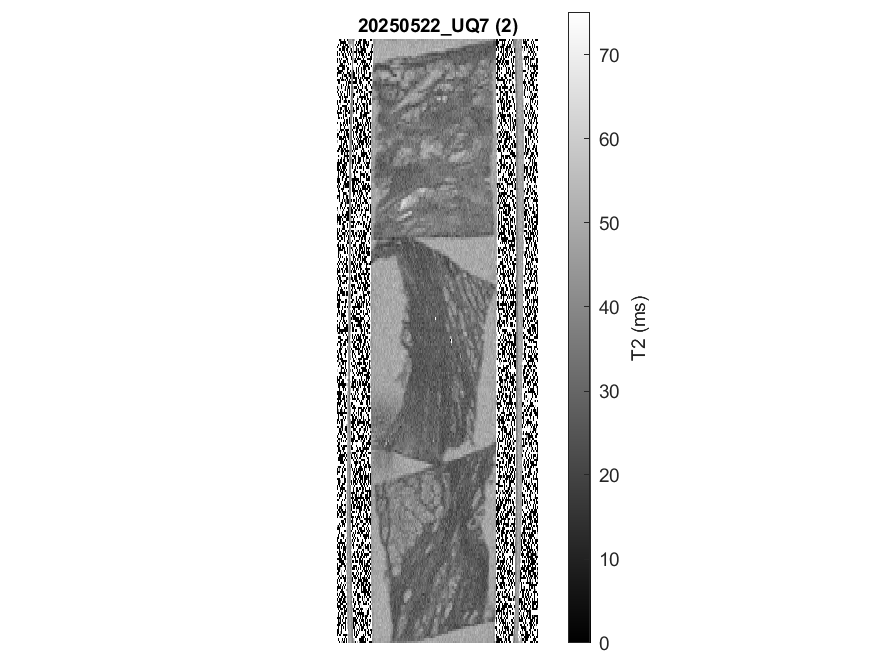


Figure S4: Sample UQ7 T2 distribution

### Sample set 5 (UQ8)

Benign, Benign, Benign (top-bottom)


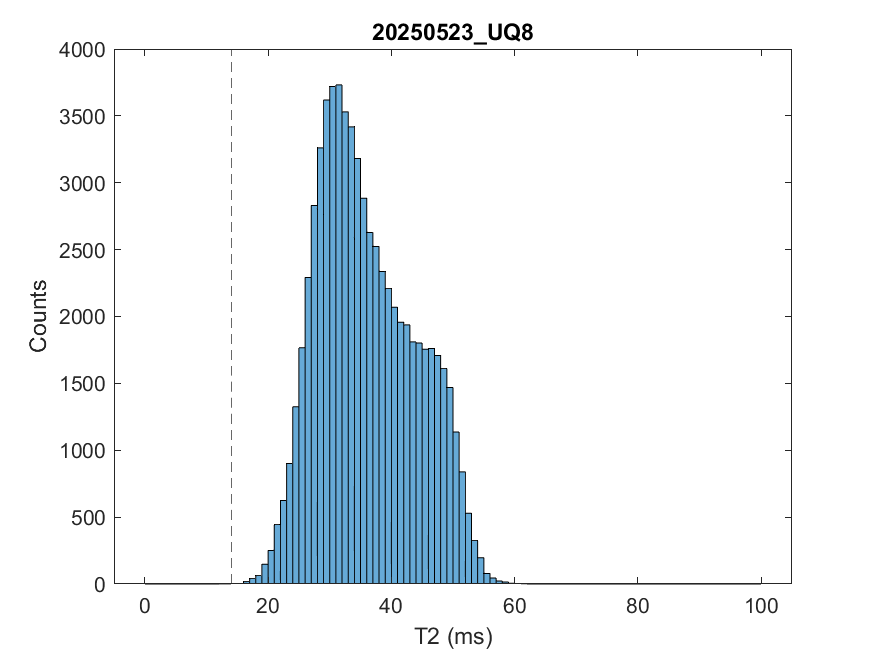

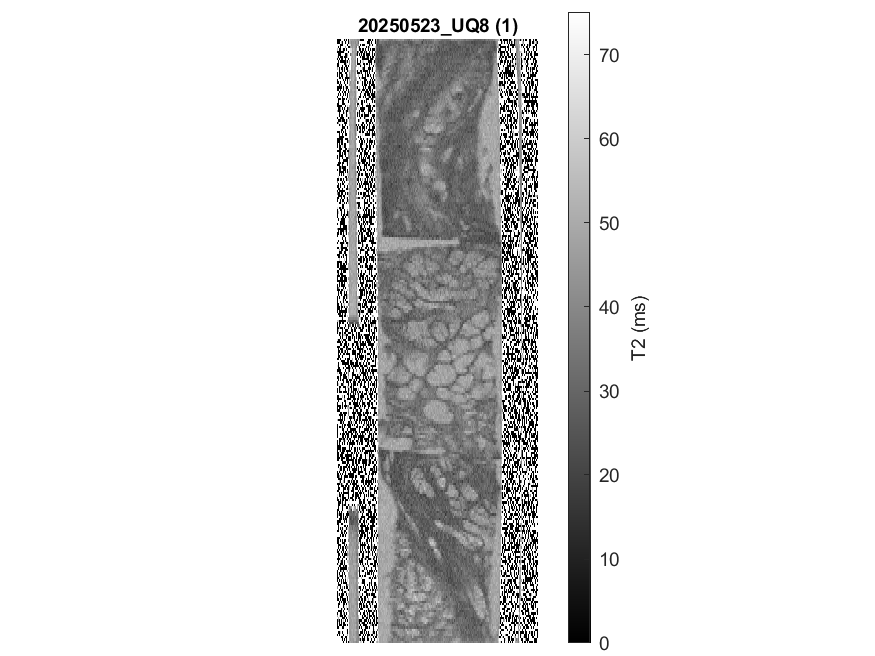

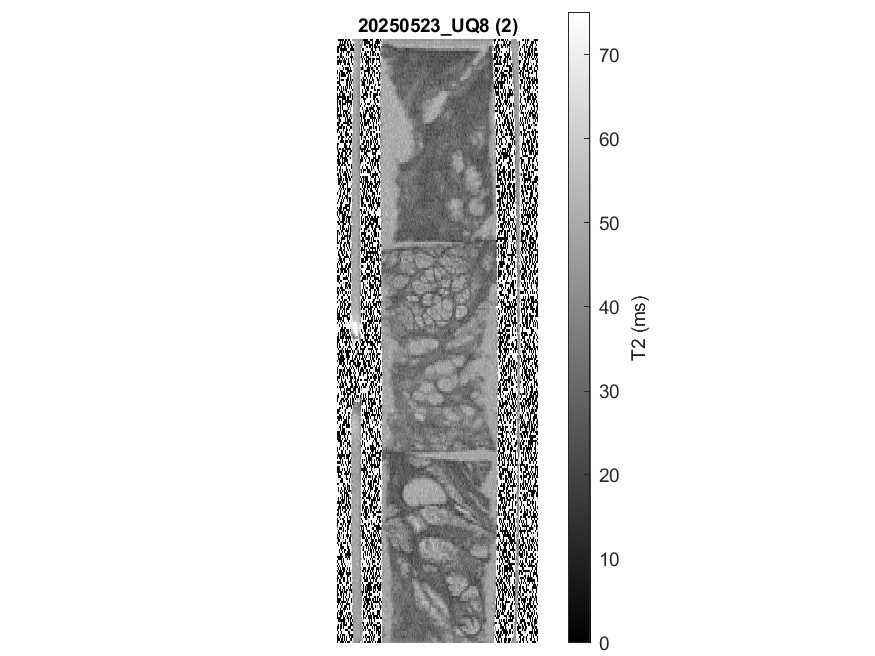


Figure S5: Sample UQ8 T2 distribution

### Sample set 6 (UQ9)

Benign, Benign (top-bottom)


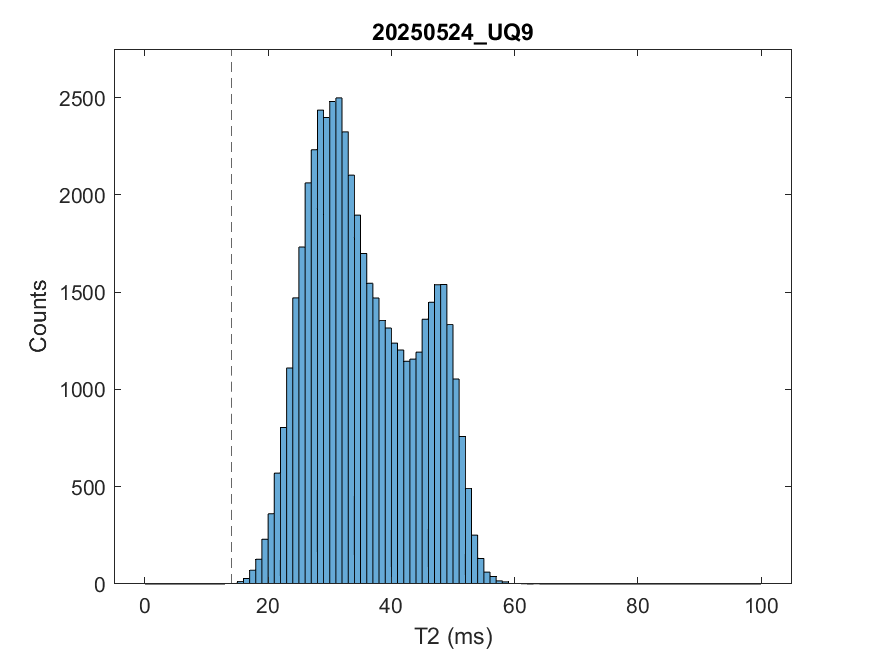

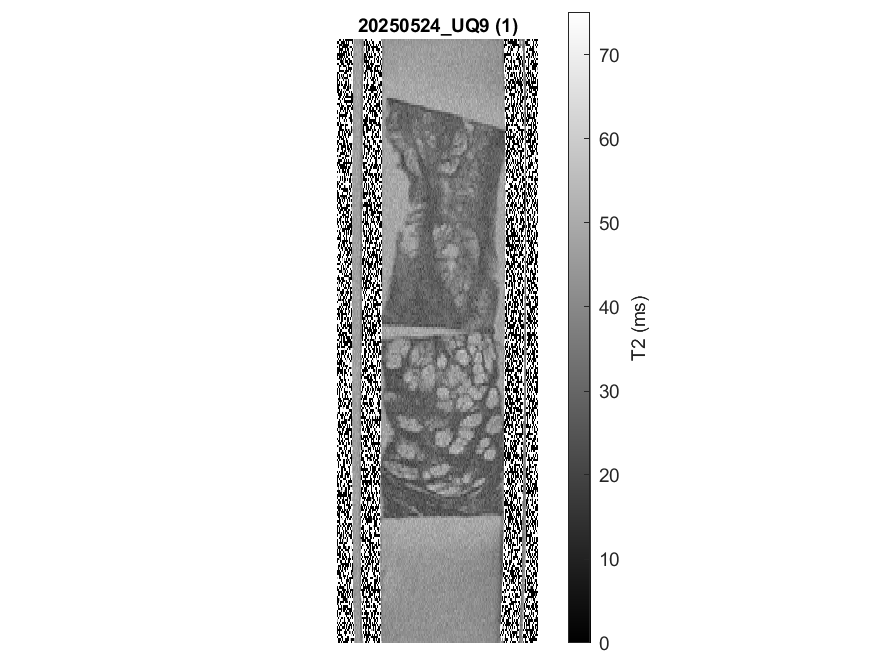

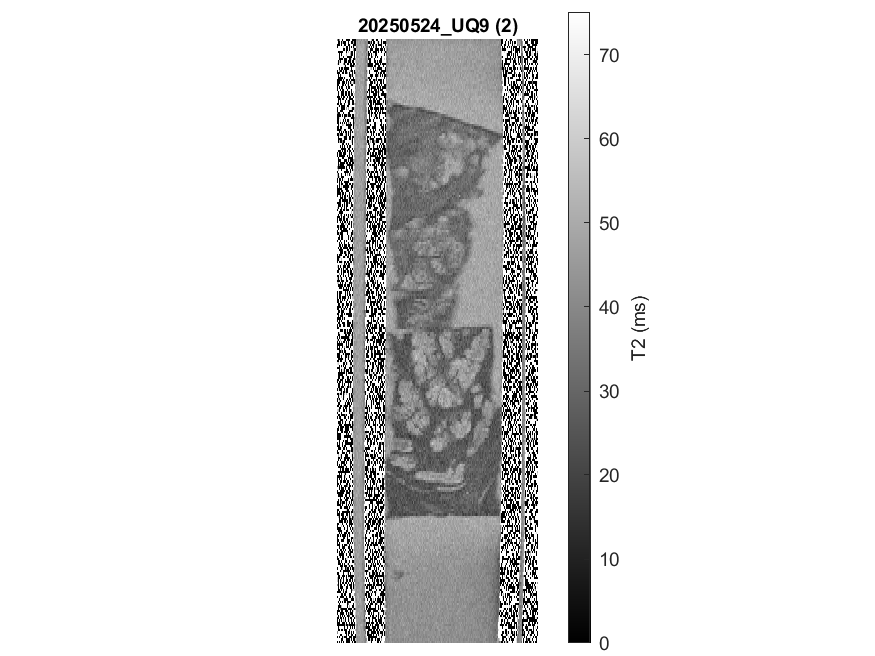


Figure S6: Sample UQ9 T2 distribution

### Comments

The medium surrounding the samples has T2≈50ms. Stromal and epithelial T2 values are ≈30ms and ≈40ms, respectively.

In this work, dMRI images with echo time TE=14ms are normalised by a b=0 image also with TE=14ms. Each voxel contains a mixture of microscopic tissue components, each with individual relaxation properties. As a result, normalisation by the voxel b=0 signal does not fully remove relaxation effects.

For a voxel containing a mixture of epithelium and stroma, the voxel signal will decay (with echo time) more slowly than the stromal signal component, but more quickly than the epithelial signal component. As a result, normalisation by the b=0 voxel signal will enhance the epithelial signal component and dampen the stromal signal component**.

With T2_E_ = 40ms, T2_S_ = 30ms, and f_E_ = f_S_ = 0.5 (equal composition of stroma and epithelium), this leads to errors of ≈5% in the normalised signal components. Errors from voxel b=0 signal normalisation are worse when a component has a small volume fraction within a voxel; however, in this case, the normalised voxel signal has a weaker influence on the aggregate signal estimate for that tissue component. Therefore, errors on aggregate signal estimates due to voxel b=0 signal normalisation are unlikely to be more than ≈5%.

Nevertheless, an improvement to this study would be to acquire the MSME sequence in alignment with dMRI sequences. The microstructure segmentations could then be used to compute aggregate T2 values for each component, and this T2 information could be included in linear model fitting to avoid normalisation error influencing aggregate dMRI signal estimates.

**

Let $T2_{vx}$ be the voxel T2 value, $T2_{E}$ be the epithelial T2 value, and $T2_{S}$ be the stromal T2 value ($T2_{S}<T2_{vx}<T2_{E}$); $f_{E}$ and $f_{S}$ are the volume fractions of epithelium and stroma within the voxel; and $ADC_{E}$ and $ADC_{S}$ are the apparent ADC values for epithelium and stroma for the specific sequence.

$$\frac{S_{b}}{S_{0}}= \frac{S_{b}^{E}+S_{b}^{S}}{S_{0}}= \frac{1}{e^{- \frac{TE}{T2_{vx}}}}\cdot\left[ {f_{E}\cdot e}^{-\frac{TE}{T2_{E}}}\cdot e^{-b\cdot ADC_{E}}+ {f_{S}\cdot e}^{-\frac{TE}{T2_{S}}}\cdot e^{-b\cdot ADC_{S}} \right]$$

$$\frac{S_{b}}{S_{0}}=\frac{e^{- \frac{TE}{T2_{E}}}}{e^{- \frac{TE}{T2_{vx}}}} \cdot{f_{E}\cdot e}^{-b\cdot ADC_{E}}+ \frac{e^{- \frac{TE}{T2_{S}}}}{e^{- \frac{TE}{T2_{vx}}}} \cdot{f_{S}\cdot e}^{-b\cdot ADC_{S}}$$

$$\frac{S_{b}}{S_{0}}=\alpha\cdot{f_{E}\cdot e}^{-b\cdot ADC_{E}}+\beta\cdot{f_{S}\cdot e}^{-b\cdot ADC_{S}}$$

With $\alpha>1$ and $\beta<1$.

## Sensitivity of results to thresholds used for microstructure segmentation

Here, we investigate the sensitivity of microstructure segmentations and aggregate signals estimates to small changes in the gradient echo and D*FA thresholds used for microstructure segmentation.

The segmentation results from the central sample of sample set 5 (UQ8) are presented due to the mixture of stroma, glands, and fluid filled spaces within this sample.

### Varying lumen/fluid threshold (MGE)

The gradient echo threshold used for segmentation of lumen/fluid was varied by ±10% for each sample set.

Microstructure segmentation was re-performed for each sample set, then aggregate epithelial and stromal signals were computed for each dMRI sequence (Section 2.3.3). Segmentation results and aggregate signal estimates are displayed in the following two figures (Figures S7 and S8).

Comments

Segmentations and aggregate signal estimates are clearly sensitive to the gradient echo threshold used for lumen/fluid segmentation.

- When the threshold is raised, many voxels in the surrounding medium and larger regions of fluid are classified as epithelium, which is clearly incorrect. The number of voxels within glands classified as lumen is reduced. As a result, aggregate epithelial signal estimates are reduced since fast-decaying signals from lumen/fluid are attributed to the epithelial component. The impact on aggregate stromal signal estimates is minimal.
- When the gradient echo threshold is lowered, many more voxels within glands are classified as lumen. In reality, these voxels likely contain a mixture of epithelium and lumen; lowering the threshold for lumen/fluid classification reduces the number voxels containing a mixture of epithelium and lumen that are classified as epithelium. Consequently, the aggregate signal estimates for epithelium are raised.

Generally, gradient echo images offered poor contrast between epithelium and lumen within glands. This could be due to partial volume effects of lumen and epithelium, or could be due to a difference in T2 between luminal fluid and the surrounding medium. We decided that the safest option for segmentation of lumen/fluid was to set the highest gradient echo threshold that correctly classified the surrounding medium and larger fluid-filled spaces as lumen/fluid and acknowledge that aggregate epithelial signal estimates are likely an underestimate of true epithelial signal. We address this as a study limitation.


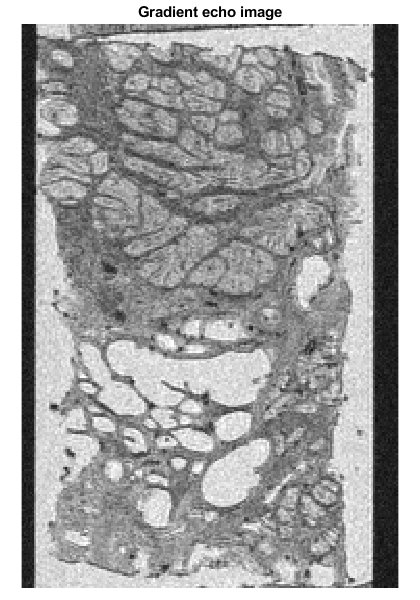

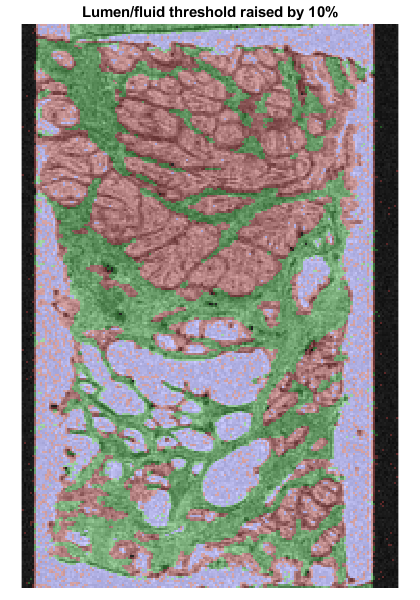

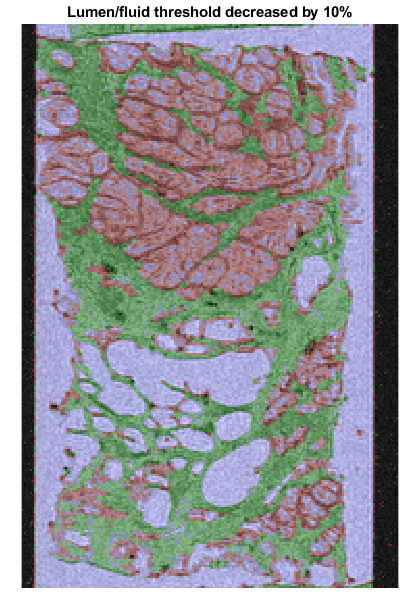

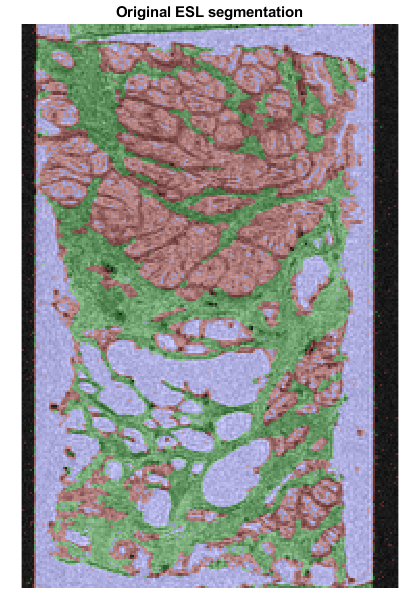


Figure S7: Microstructure segmentations following changes to the gradient echo signal threshold.


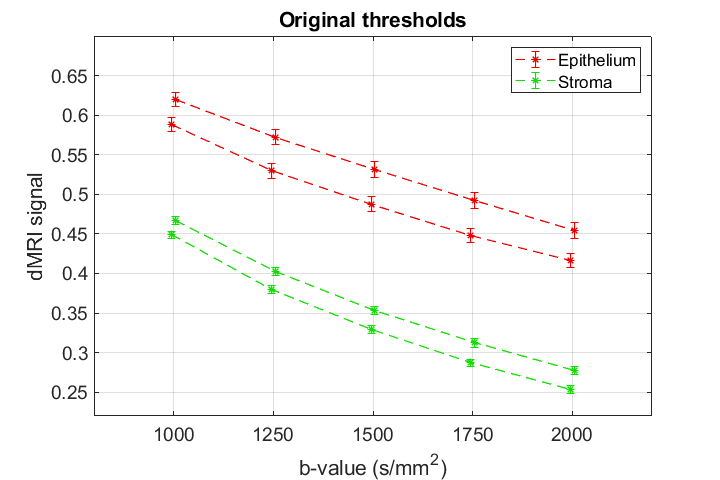

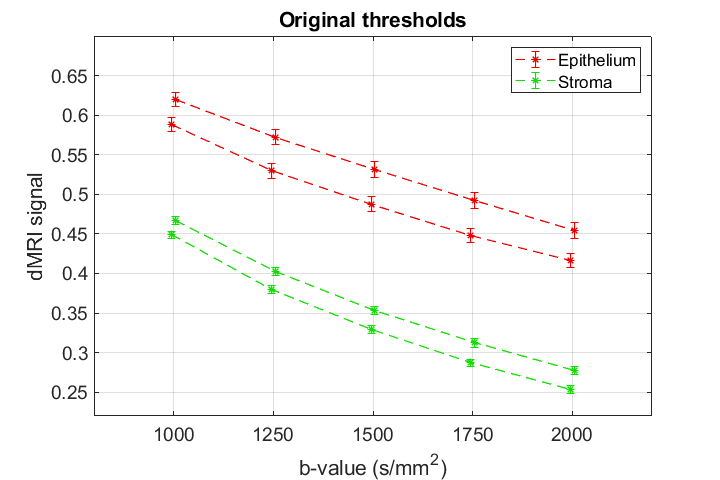

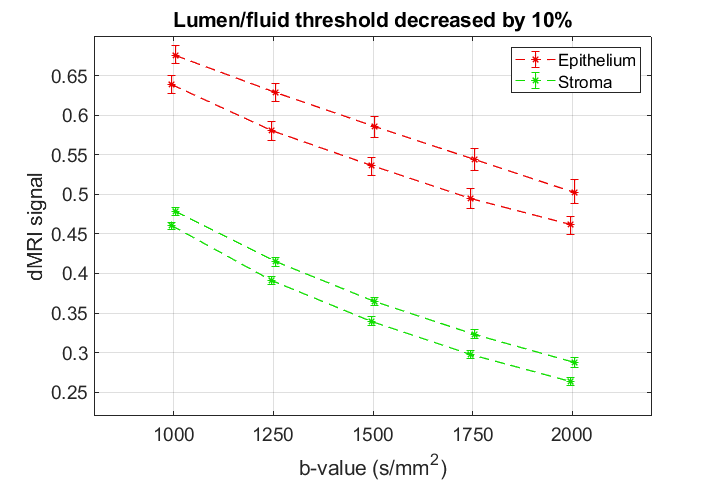

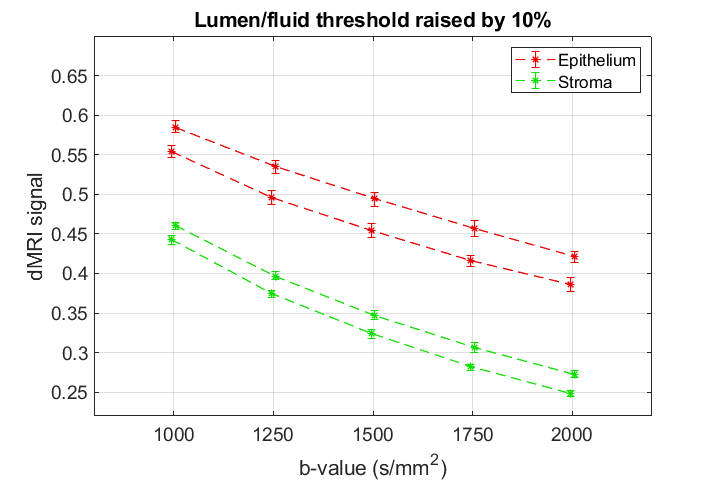


Figure S8: Aggregate epithelial and stromal signal estimates following changes to gradient echo signal threshold.

### Varying stroma threshold (D*FA)

The D*FA threshold used for segmentation of stroma was varied by ±10%.

Microstructure segmentation was re-performed for each sample set, then aggregate epithelial and stromal signals were computed for each dMRI sequence (Section 2.3.3). Segmentation results and aggregate signal estimates are displayed in the following two figures (Figures S9 and S10).

Comments

Segmentations and aggregate signal estimates are relatively robust to small changes in the D*FA threshold due to the distinct contrast between stromal and non-stromal regions (glands + regions of fluid). The stromal segmentations are consistent with the microstructure seen in histology so we are confident that they are an accurate representation of each sample’s stromal microstructure.


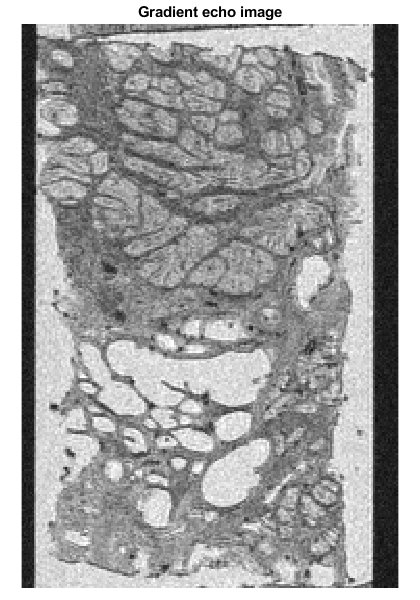

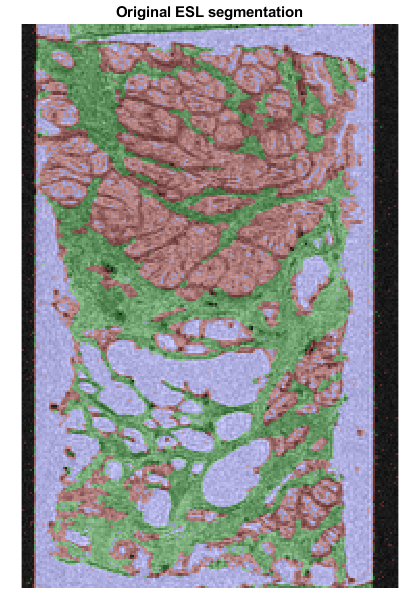

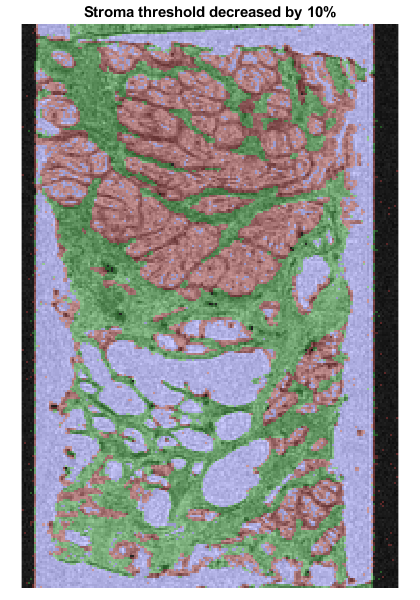

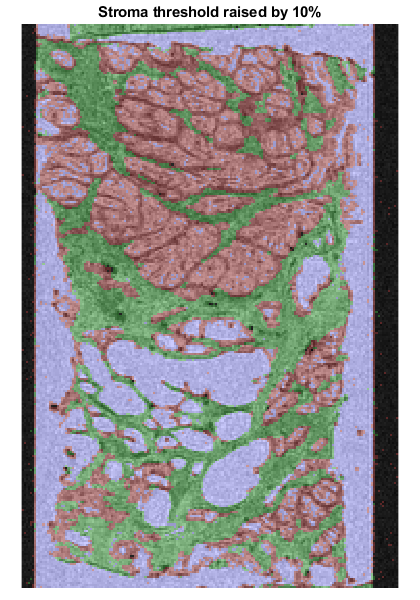


Figure S9: Microstructure segmentations following change to D*FA threshold


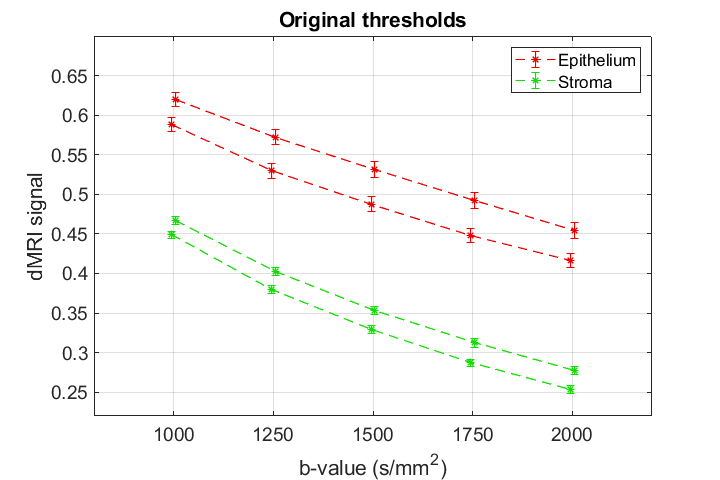

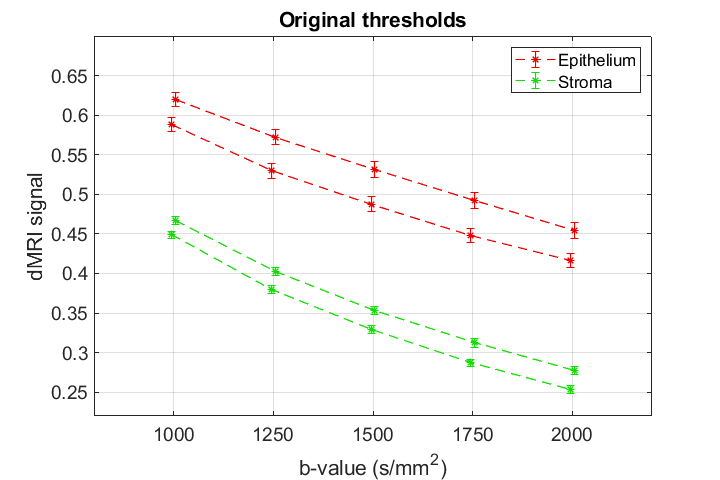

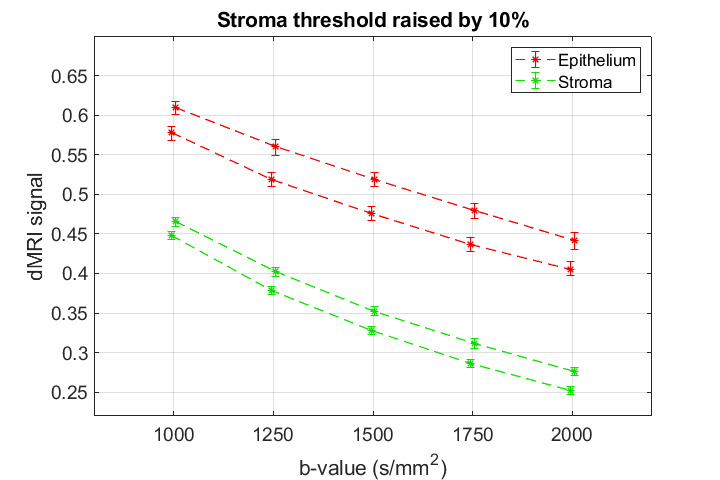

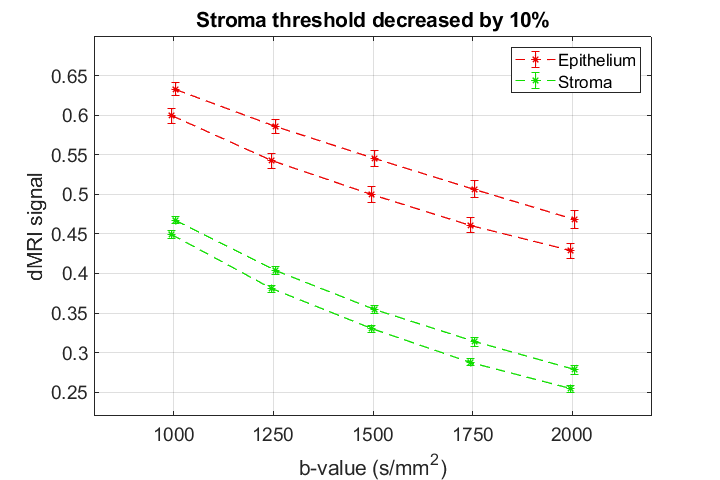


Figure S10: Aggregate epithelial and stromal signal estimates following changes to D*FA threshold

## ESL volume fraction distributions

Figures S11-13 present the volume fractions of epithelium, stroma, and lumen across all the low-resolution (480x480x320µm) voxels used for aggregate dMRI signal estimation.


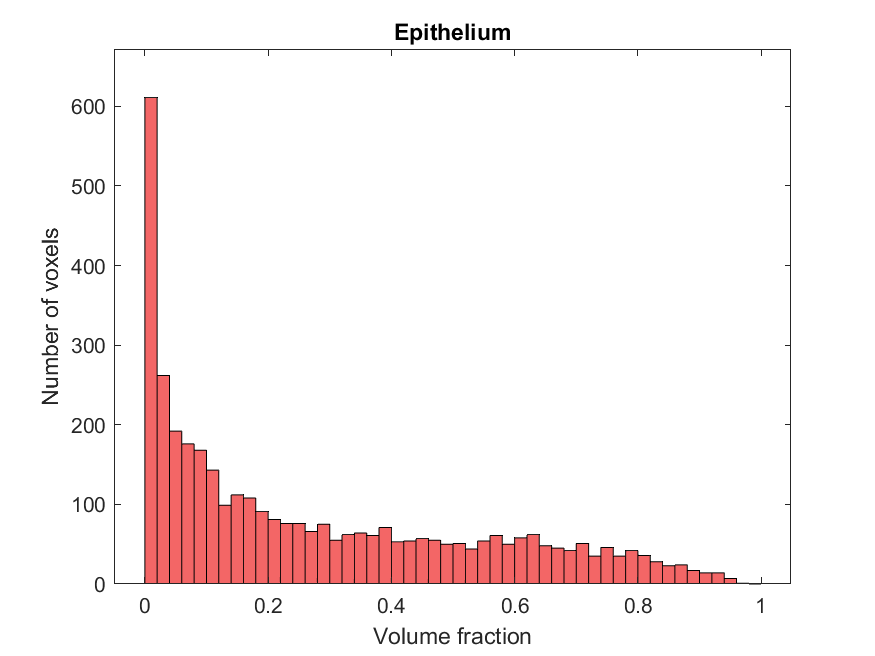


Figure S11: Epithelial volume fractions across all voxels used in analysis


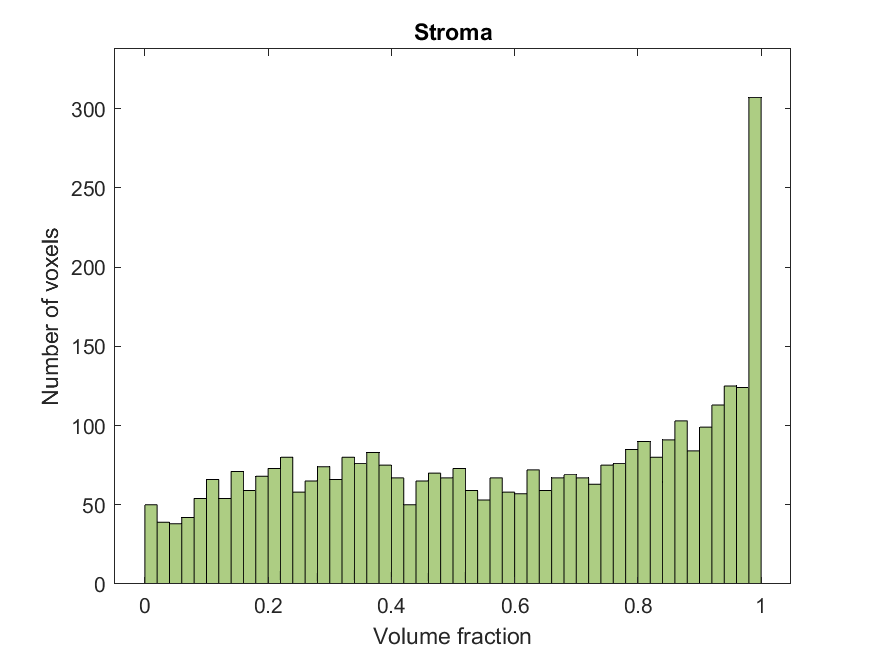


Figure S12: Stromal volume fractions across all voxels used in analysis


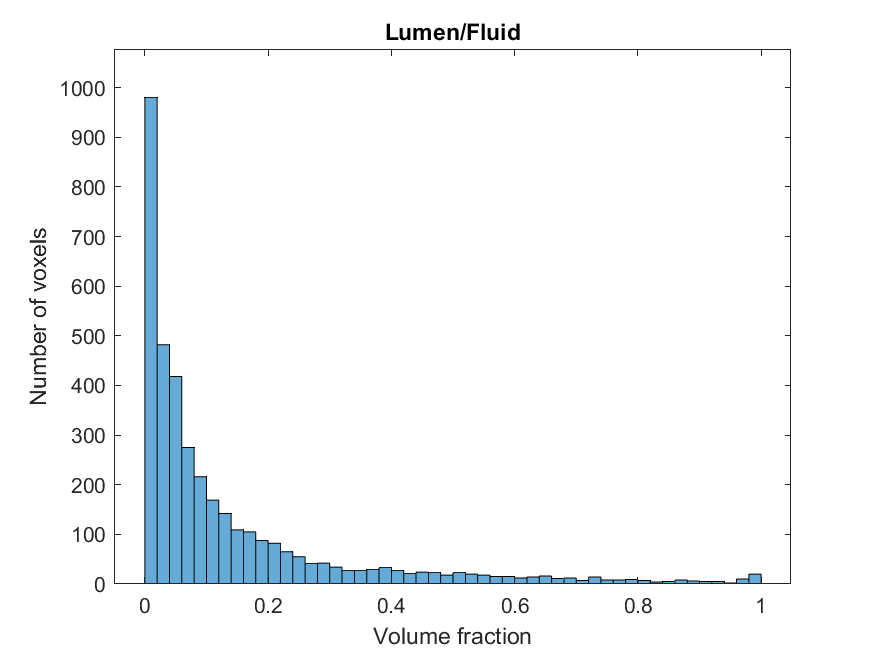


Figure S13: Lumen/fluid volume fractions across all voxels used in analysis

## Comments

The distribution of epithelial volume fractions spans most of the [0, 1] range, however there is an overall bias towards lower values, and extreme values close to 1 are not represented.

The distribution of stromal volume fractions shows full coverage of the [0, 1] range, but values are biased towards higher values.

The luminal/fluid volume fractions are strongly biased towards lower values and coverage of larger values is minimal. Inspecting the microstructure segmentations indicates that the lumen/fluid component predominantly originates from larger fluid-filled spaces and the surrounding medium, for which the diffusivity is known and confirmed through DTI processing.

These observations support the use of a two variable linear model, in which epithelial and stromal signals are estimated from the dMRI measurement data, and luminal/fluid signal is modelled using a monoexponential decay model with fixed diffusivity.

There is sufficient varied data to reliably obtain aggregate epithelial and stromal signal estimates (as indicated by the small confidence intervals obtained from bootstrapping), and this approach avoids potential fitting instability arising from the poorly sampled luminal/fluid volume fraction.

## MP-PCA denoising on DTI images

Here, we showcase the improvement to DTI image quality from MP-PCA denoising (patch size = [5,5]). Original and denoised images are displayed for two DTI directions from two sample sets (Figures S14 and S15).

The noise variance estimates returned from MP-PCA denoising place the SNR of individual directions at ≈5-10 (SNR calculated as S_DN_ / sqrt(σ^2^), where S_DN_ is the denoised signal and σ^2^ is the noise variance estimate from MP-PCA).

The improvement to image quality is clear and greatly improved the appearance of parameter maps estimated from DTI processing.


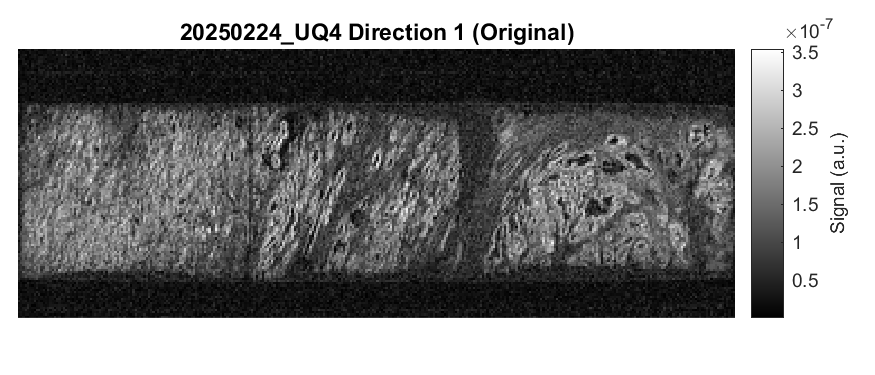

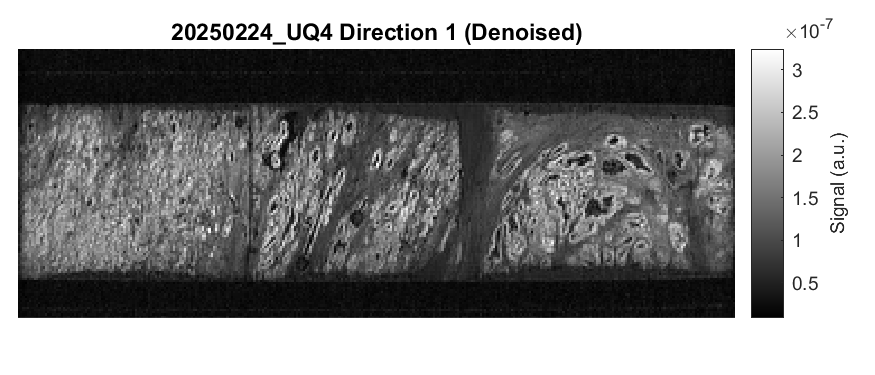

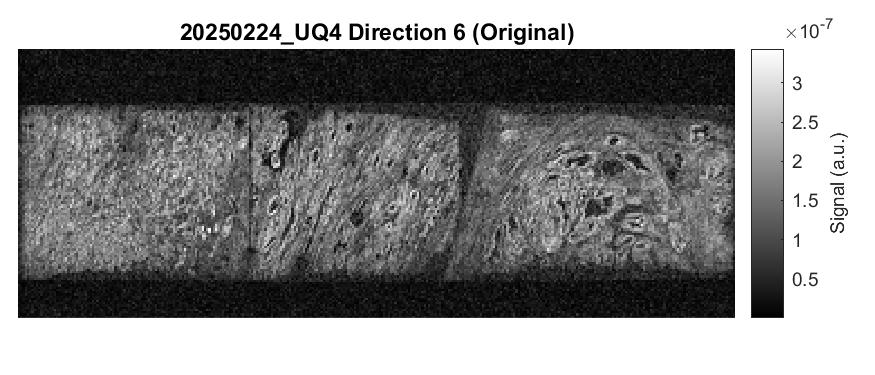

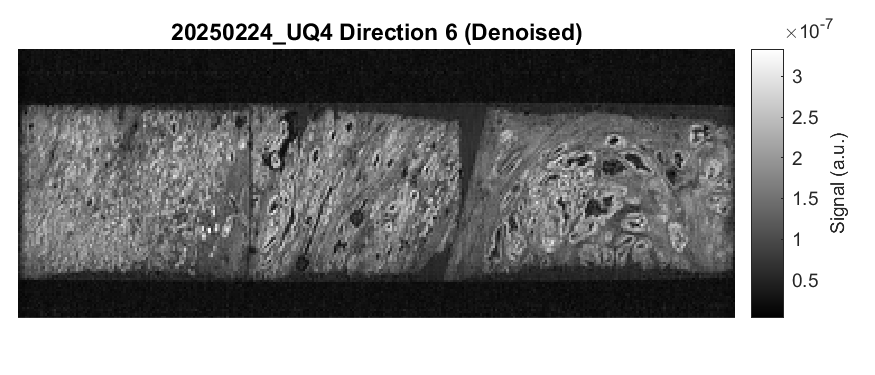


Figure S14: MP-PCA denoising results on sample UQ4


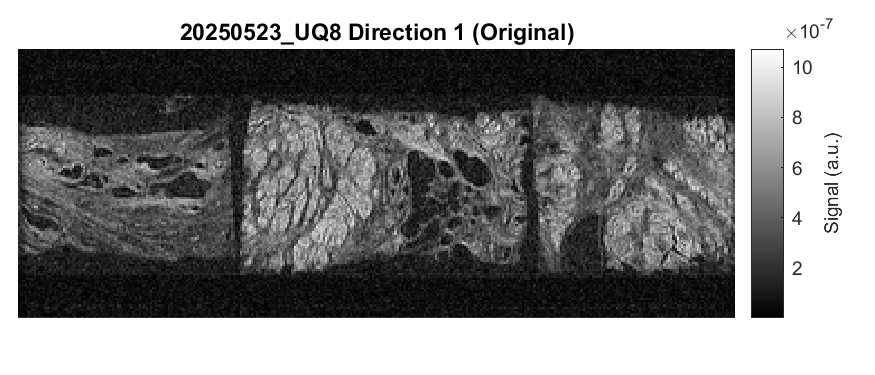

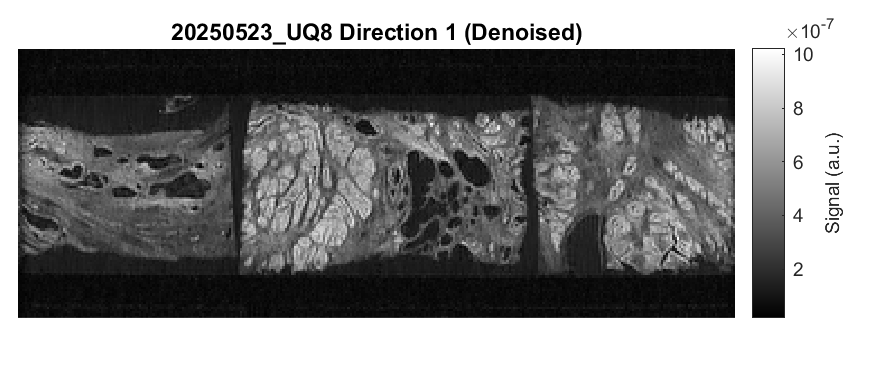

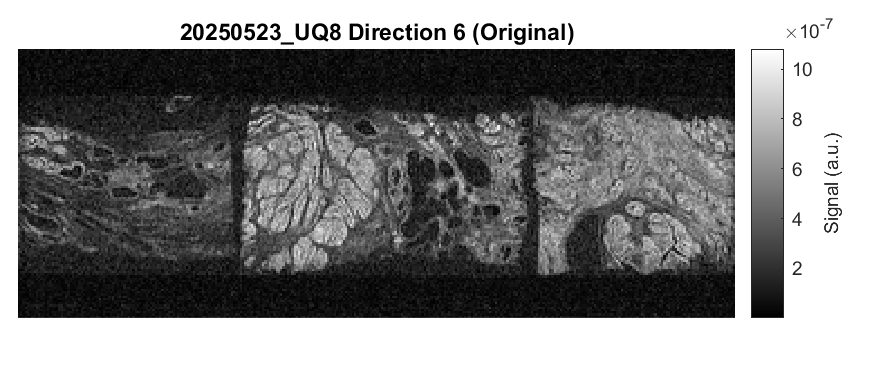

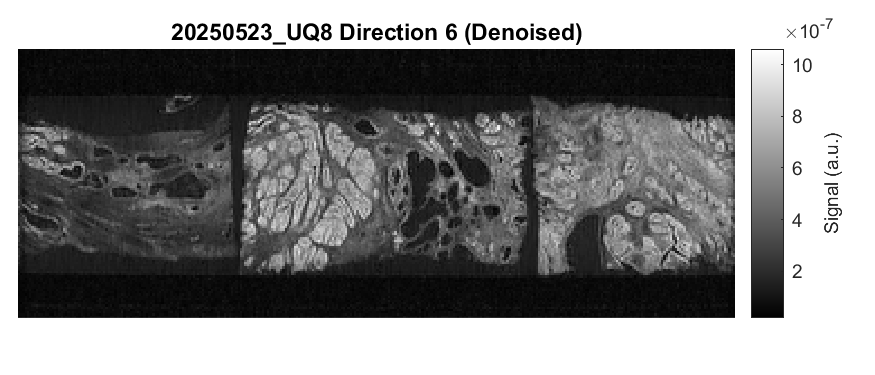


Figure S15: MP-PCA denoising results on sample UQ8

## MP-PCA denoising on multidimensional dMRI images

Here, we present the results of MP-PCA denoising on multidimensional dMRI images with b=2000s/mm^2^ and Δ=40ms (patch size = [5,5]). Original and denoised images are displayed for three directions from two sample sets (Figures S16-21).

The noise variance estimates from MP-PCA place the SNR of signal measurements from each direction at: ≈2 for voxels with high fluid/lumen content, ≈5-10 for voxels with high stromal content (direction-dependent due to stromal anisotropy), and ≈10-20 for voxels with high epithelial content. (SNR calculated as S_DN_ / sqrt(σ^2^), where S_DN_ is the denoised signal and σ^2^ is the noise variance estimate from MP-PCA).

The impact of MP-PCA denoising on dMRI images is minimal, likely because each patch spans a relatively large tissue region compared with DTI denoising, resulting in increased heterogeneity and potentially unreliable noise variance estimates (noise likely overestimated).


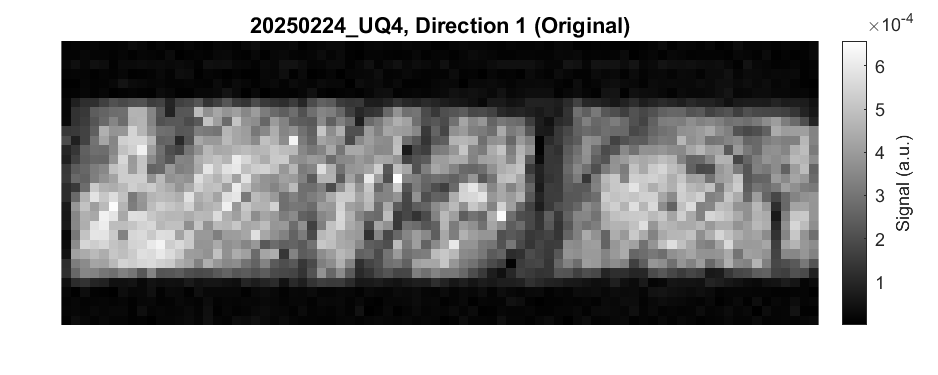

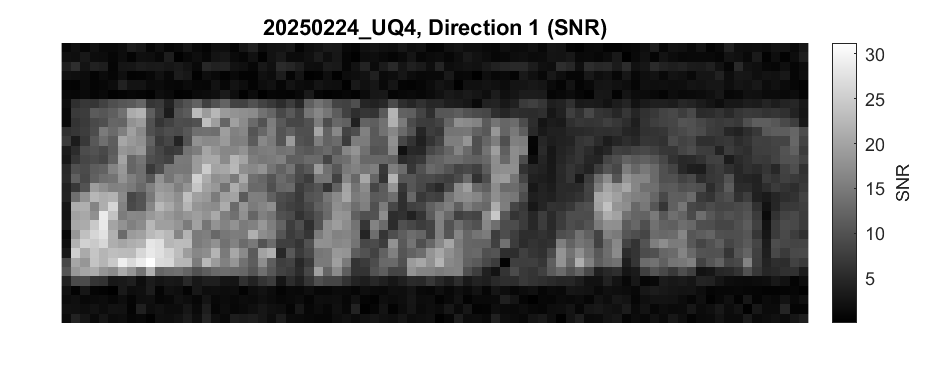

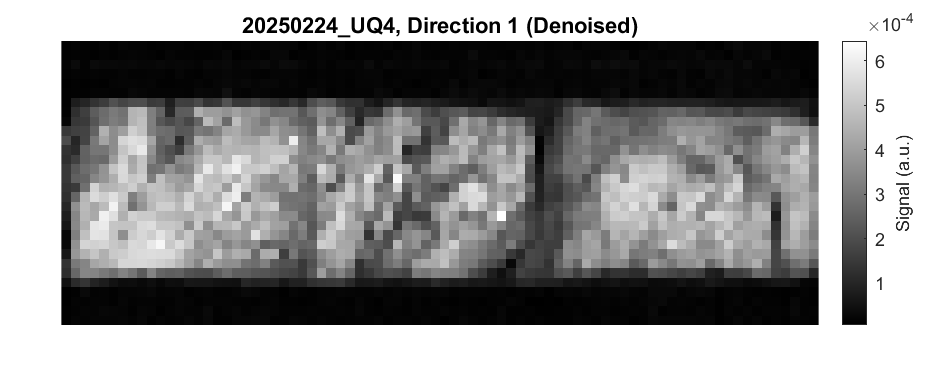


Figure S16: MP-PCA denoising on b=2000s/mm^2^, Δ=40ms dMRI image (diffusion encoding direction 1) for sample UQ4


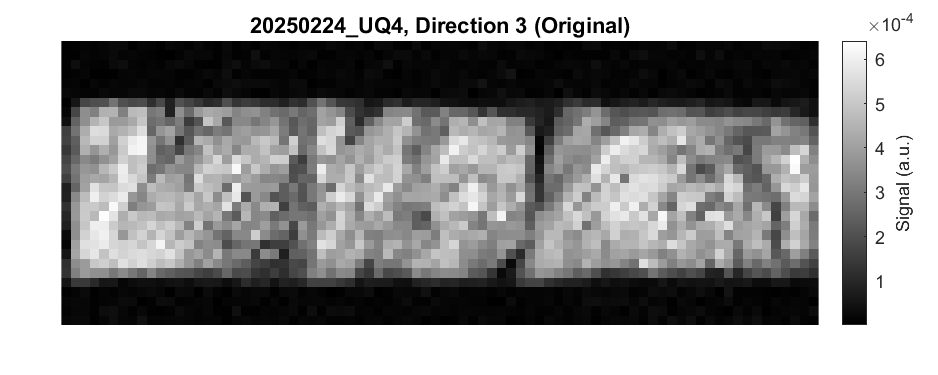

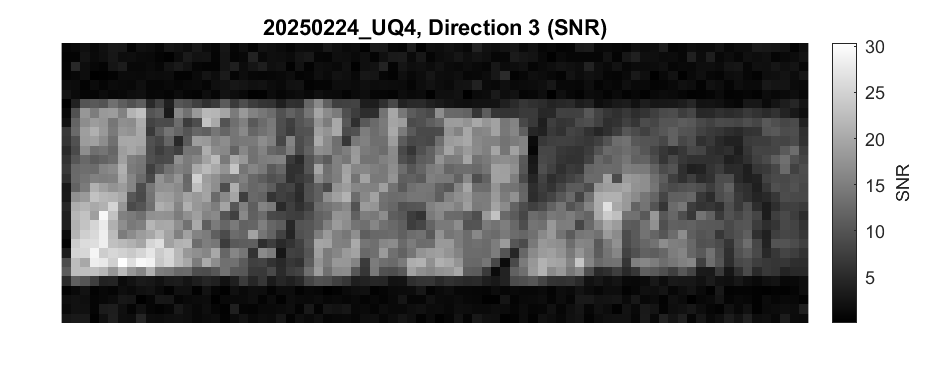

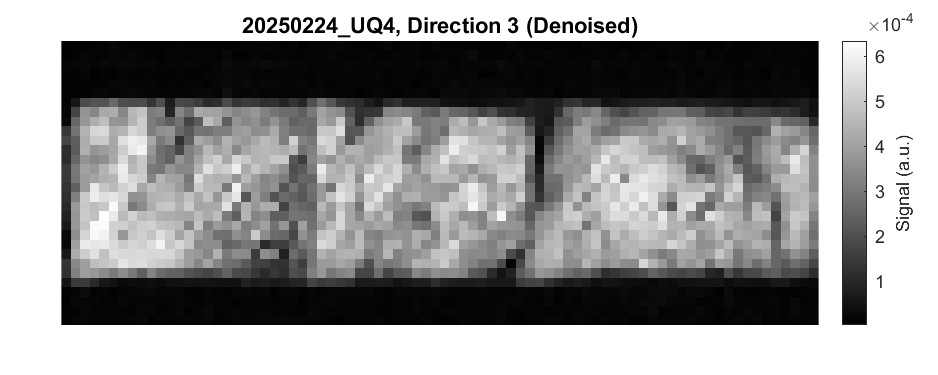


Figure S17: MP-PCA denoising on b=2000s/mm^2^, Δ=40ms dMRI image (diffusion encoding direction 3) for sample UQ4


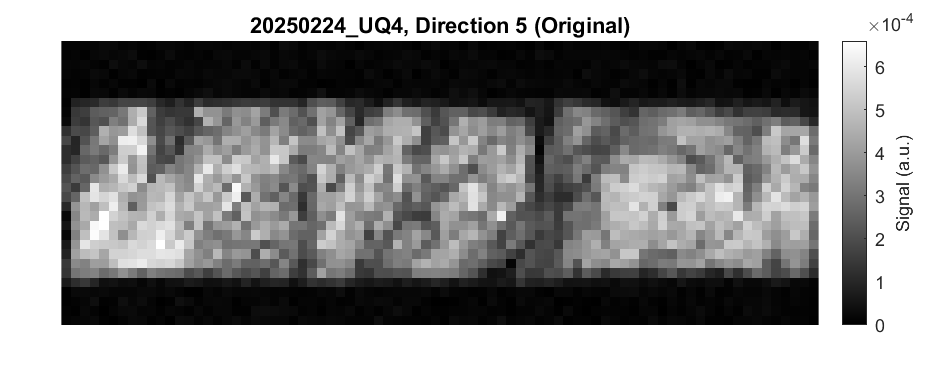

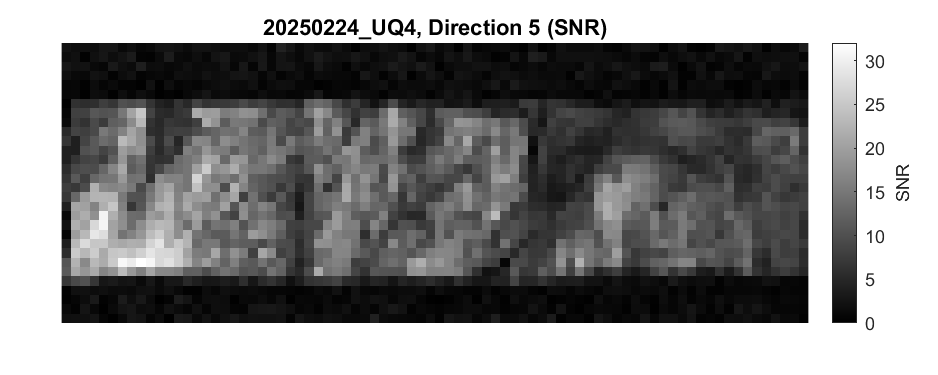

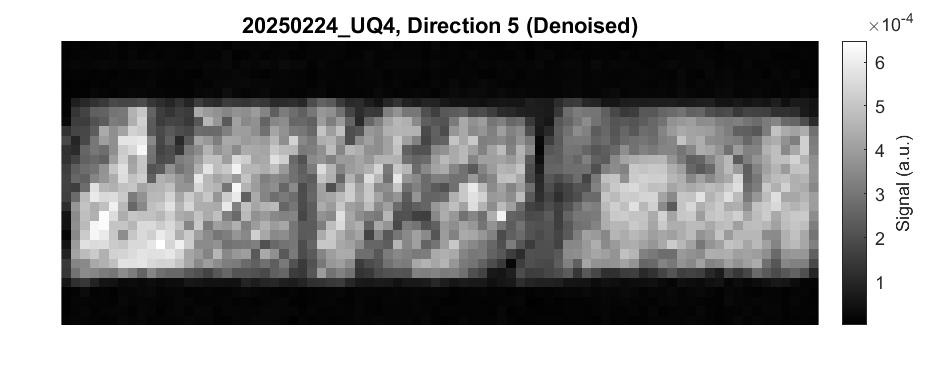


Figure S18: MP-PCA denoising on b=2000s/mm^2^, Δ=40ms dMRI image (diffusion encoding direction 5) for sample UQ4


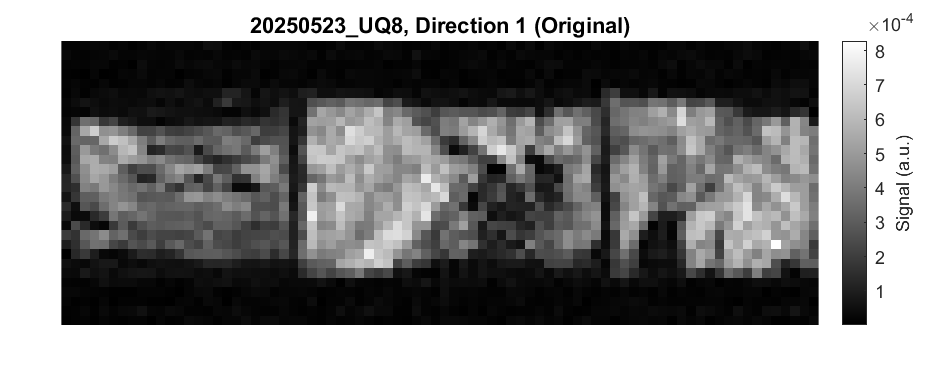

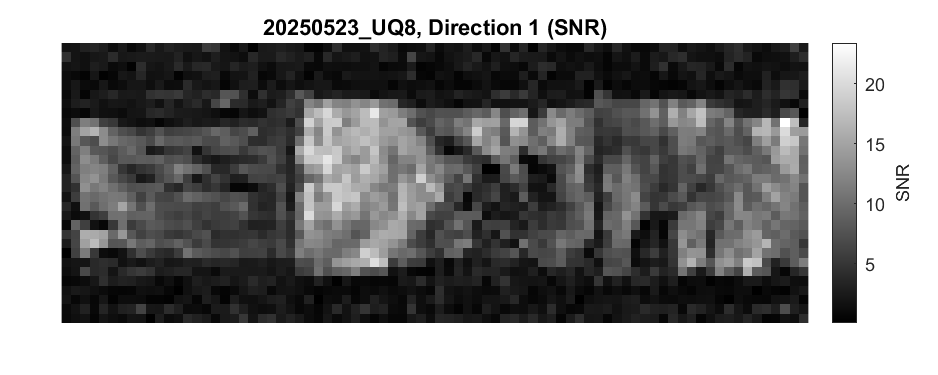

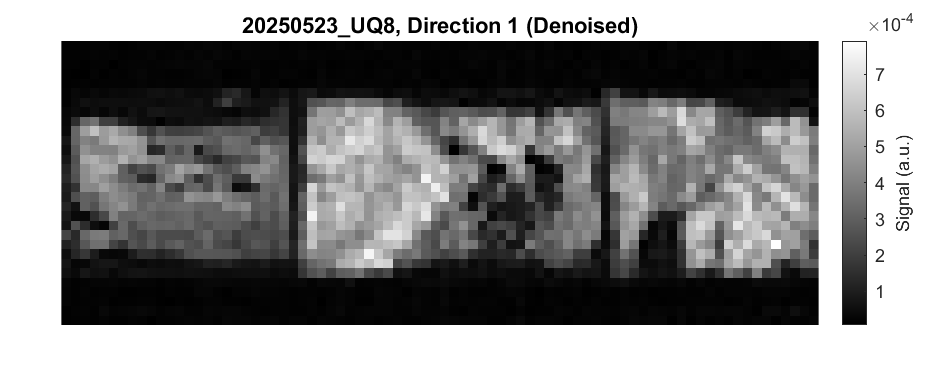


Figure S19: MP-PCA denoising on b=2000s/mm^2^, Δ=40ms dMRI image (diffusion encoding direction 1) for sample UQ8


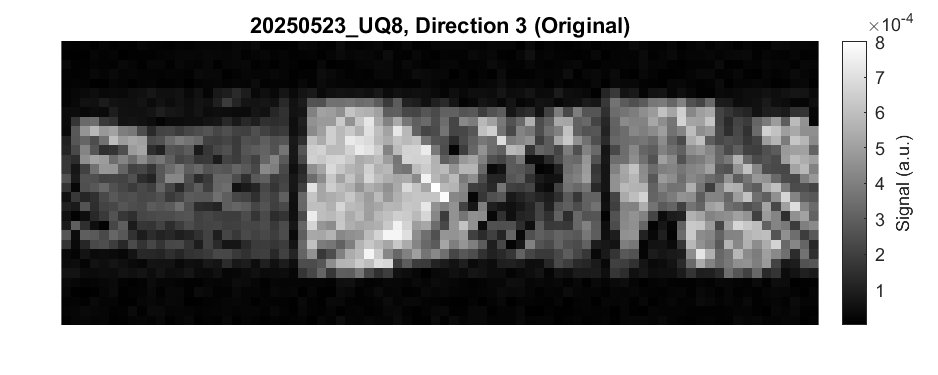

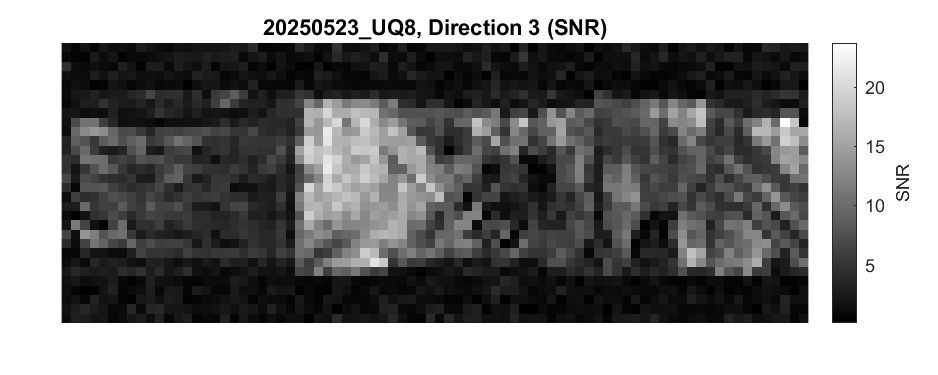

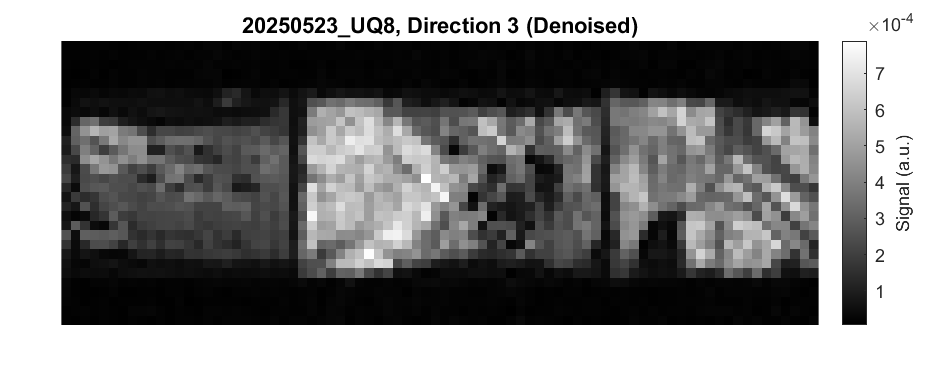


Figure S20: MP-PCA denoising on b=2000s/mm^2^, Δ=40ms dMRI image (diffusion encoding direction 3) for sample UQ8


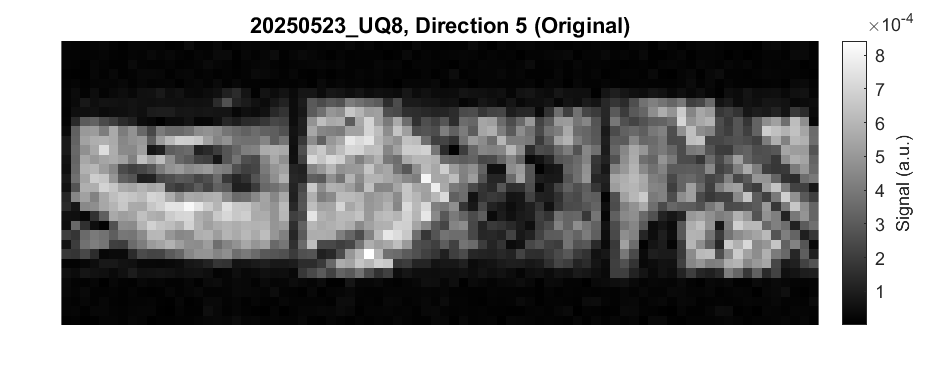

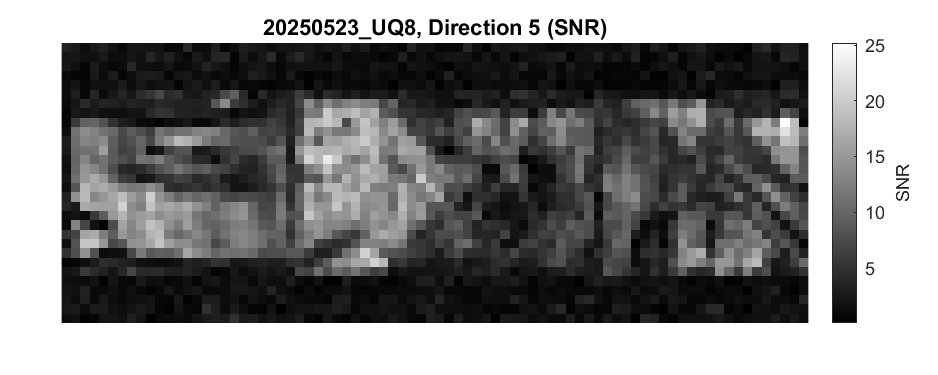

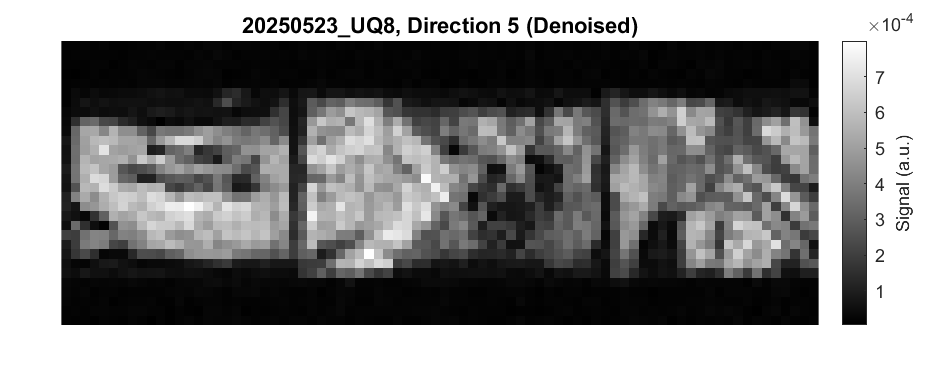


Figure S21: MP-PCA denoising on b=2000s/mm^2^, Δ=40ms dMRI image (diffusion encoding direction 5) for sample UQ8

## Stromal anisotropy effects on direction-averaged signals

Here, we investigate the potential impact of stromal anisotropy on direction-averaged dMRI signals.

The diffusion tensor in stromal fibres was approximated with two diffusivities: D_para_ , diffusivity parallel to fibre direction; and D_perp_ , diffusivity perpendicular to fibre direction.

Within a voxel, stroma could:

1. form a single band with aligned fibre directions
2. consist of multiple sub-voxel regions with varying fibre directions

Through simulation, we explore how these different cases affect the direction-averaged dMRI signal measured from the voxel.

From DTI processing results, we find that typical diffusivities in bands of stroma are

- D_para_ = 1.5 µm^2^/ms
- D_perp_ = 0.5 µm^2^/ms

Figure S22 displays example maps of D_para_ and D_perp_ from sample UQ4.


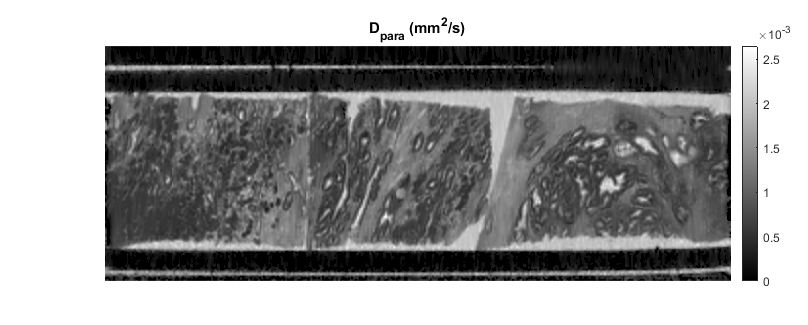

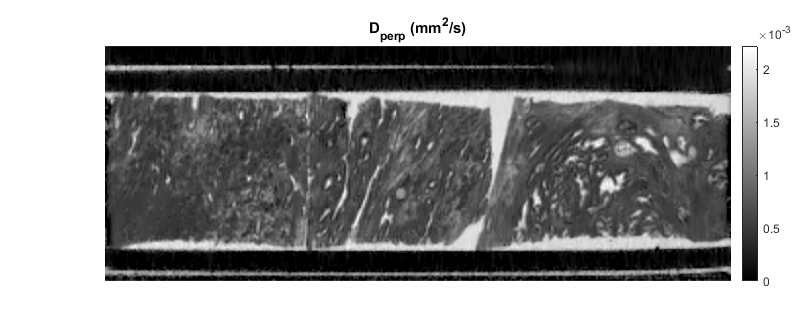


Figure S22: Parallel and perpendicular diffusivity estimates from DTI processing (UQ4)

The direction-averaged dMRI signal (over the six gradient directions used in dMRI sequences) was simulated for two cases:

1. Voxel containing single band of aligned stromal fibres (uniform diffusion tensor across voxel)
2. Voxel containing three separate bands of stromal fibres each with randomised fibre direction (distinct diffusion tensor in each band)

Simulation for case 2 were repeated N=1000 times with randomised fibre directions each time.

Simulations were performed for b-values [1000, 1250, 1500, 1750, 2000] s/mm^2^, as used in multidimensional dMRI.

Simulations were also repeated for more highly anisotropic diffusion in stromal fibres: D_perp_ = 0.4, 0.3, 0.2 µm^2^/ms. This approximately simulates impact of longer diffusion times (lower apparent diffusivity in directions perpendicular to fibre direction).

The MATLAB code used for simulation is provided in the script: invest_anisotropy_effects.m (See GitHub reference)

Results

Figures S23-26 display the results from simulation. The blue asterisk markers represent direction-averaged signals from voxel 1 (single band of aligned stromal fibres). These markers are offset to the right for visual clarity (avoiding overlap with boxplots). Boxplots show the distributions of direction-averaged signals when directions of sub-voxel stromal bands are randomised. The boxplot labels display the mean and standard deviation of these distributions.


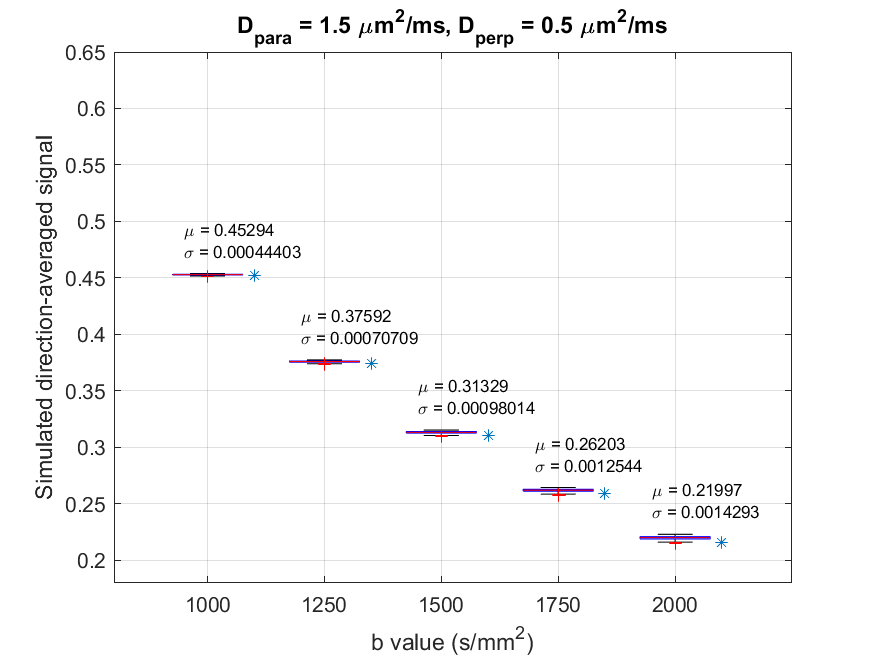


Figure S23: Simulated direction-averaged dMRI signals for D_perp_=0.5 µm^2^/ms.


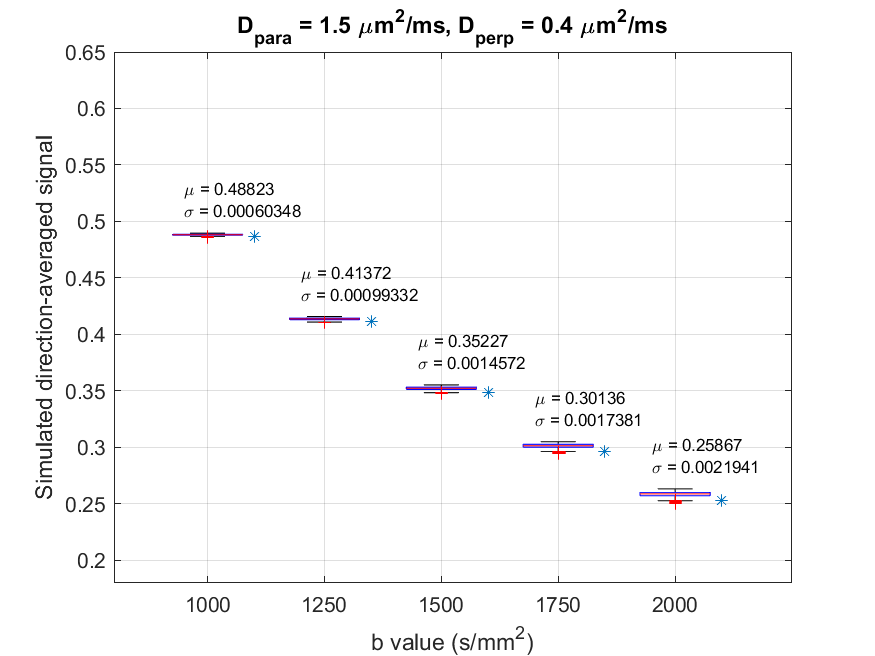


Figure S24: Simulated direction-averaged dMRI signals for D_perp_=0.4 µm^2^/ms.


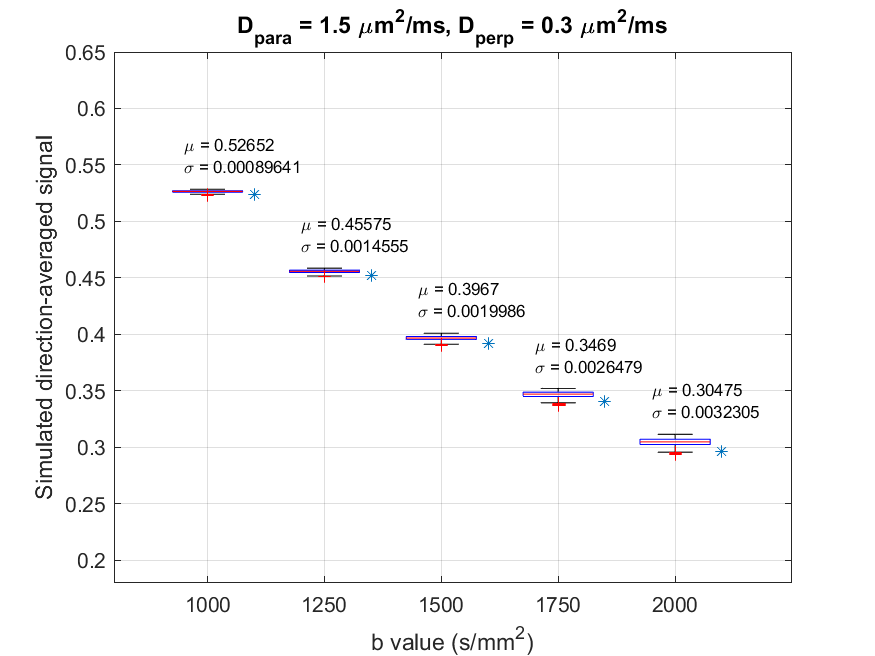


Figure S25: Simulated direction-averaged dMRI signals for D_perp_=0.3 µm^2^/ms.


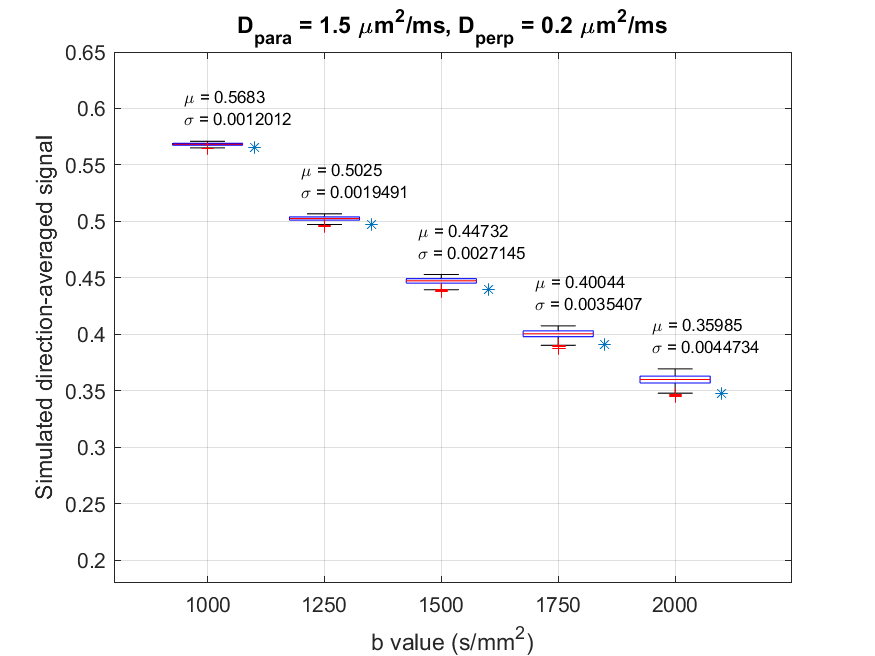


Figure S26 Simulated direction-averaged dMRI signals for D_perp_=0.4 µm^2^/ms.

Comments

The standard deviation of direction-averaged signals increases for more highly anisotropic stromal diffusion (smaller D_perp_), and is larger for the higher b-value sequences.

The simulation results for D_perp_ = 0.4 µm^2^/ms are most similar to the aggregate stromal signal estimates at longer diffusion times (Δ^+^).

In all cases, variation in direction-averaged signals is small (σ<0.01). This is far smaller than the residual variation between measured and predicted signals observed for voxels with high stromal content. As such, signal variability due to residual anisotropy effects is likely to be very small, though will have a slightly larger impact for sequences with higher b-value and longer ∆.
